# Electric Field Driven Soft Morphing Matter

**DOI:** 10.1002/adma.202419077

**Published:** 2025-06-12

**Authors:** Ciqun Xu, Charl F. J. Faul, Majid Taghavi, Jonathan Rossiter

**Affiliations:** ^1^ School of Engineering Mathematics and Technology University of Bristol Bristol BS8 1TW UK; ^2^ Bristol Robotics Laboratory Bristol BS16 1QY UK; ^3^ School of Chemistry University of Bristol Bristol BS8 1TS UK; ^4^ Department of Bioengineering Imperial College London London SW7 2AZ UK; ^5^ School of Engineering and Materials Science Queen Mary University of London London E1 4NS UK

**Keywords:** electric field actuation, electroactive material, shape‐morphing gel, bio‐inspired actuation, soft robots

## Abstract

The manipulation of soft morphing robots using external electric fields and wireless control is challenging. Electric field‐driven soft morphing matter, termed electro‐morphing gel (e‐MG), that exhibits complex multimodal large‐scale deformation (showing up to 286% strain, and strain rates up to 500% s^−1^) and locomotion under external electric fields applied using compact and lightweight electrodes is presented. The distinctive capabilities of e‐MG derive from the combination of an elastomeric matrix and nanoparticulate paracrystalline carbon. The material properties, electroactive principle, and control strategies are explored and demonstrate fundamental morphing matter behaviors including rotating, translating, stretching, spreading, bending, and twisting. A range of potential bio‐inspired applications, including slim mold‐like spreading, snail‐like jumping over a gap, object transport, wall climbing, and a frog tongue‐inspired gripper is shown. The e‐MG provides morphing capabilities beyond the current limitations in wireless control for a wide range of applications in soft and bio‐inspired robotics, dexterous manipulation, and space exploration.

## Graphical abstract

This study presents soft morphing matter robots that exhibit extremely high morphological adaptability and complex multimodal electric field response. They can undergo large, controlled body deformation and show complex locomotion on different terrains under the manipulation of external electric fields, making a significant step beyond current electroactive and soft robots.

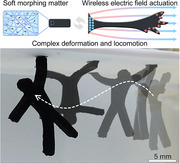

## 1 Introduction

Soft morphing matter is an emerging class of robots that are composed of soft materials and capable of generating intricate and reversible body morphing under wireless actuation. Their transformability and functionality enable them to extend the boundaries of conventional robots, providing unique solutions for a wide range of applications in industry, engineering, wearable devices, and healthcare.^[^ ^1^, ^2^, ^3^, ^4^, ^5^, ^6^ ^]^ Wireless control methods release the soft morphing matter robots from the constraints and limitations posed by external connections, and enhance mobility, dexterity, and adaptability, creating possibilities for applications where tethered systems are infeasible.^[^ ^1^, ^7^ ^]^ As one of the most investigated remote actuation methods, magnetic field actuation allows robots to realize multimodal locomotion and myriad morphing patterns.^[^ ^8^, ^9^, ^10^, ^11^ ^]^ However, precise control requires complex, bulky, and power‐consuming systems while offering only a restricted range of motion. Tether‐free actuation driven by light, heat, and acoustics has also been demonstrated^[^ ^6^, ^12^, ^13^, ^14^, ^15^ ^],^ but their wireless controllable morphing suffers from shortcomings, with none of the current methods delivering high response speed, complex shape‐shifting, and independent manipulation.

Electric field‐driven wireless actuation offers the possibility to realize sophisticated soft morphing robots by exploiting complex, controllable, and reconfigurable electric fields established using lightweight and low‐cost electrodes. Traditionally, untethered electric actuation is realized by hydrogels that contain polyelectrolyte networks with fixed charges and mobile ions.^[^ ^16^, ^17^, ^18^ ^]^ The migration of ions in response to an applied electric field induces swelling or contracting of the hydrogel, resulting in movement and deformation. Although strategies have been employed to enhance their performance, ionic hydrogels suffer from low actuation speed and limited morphing complexity. Other electroactive materials have been proposed for development into soft matter,^[^ ^19^, ^20^, ^21^, ^22^, ^23^, ^24^, ^25^, ^26^ ^]^ such as dielectric elastomer actuators, but these typically rely on the generation of internal electric fields and are built with on‐body electrodes, which compromise deformability, versatility, and adaptability. Despite generating high response speed and force, dielectric elastomers must be tethered to an external power supply – precluding wireless control – or be driven by bulky on‐board batteries and control circuitry. The development of responsive soft matter capable of controllable and varied morphing under contactless electric field stimulation remains challenging.

Here we address this challenge and present the electro‐morphing gel (e‐MG) that exhibits large‐scale deformation and locomotion, with multiple and complex morphologies, under external electric fields. Morphing, locomotion, and object moving are accomplished by controlled electrical stimulation of remote planar and cylindrical electrodes, establishing non‐uniform electric fields that drive both local and global mobility and metamorphosis through complementary dielectrophoretic and charge‐coupled effects. The underlying electromechanical transduction mechanisms in different media including air and dielectric liquid, are investigated. The e‐MG can accomplish sophisticated and challenging tasks via bio‐inspired strategies such as snail‐like jumping over a gap and frog tongue‐like gripping, demonstrating their potential for myriad industrial, biomedical, and aerospace applications.

## 2 Results

### 2.1 Electro‐Morphing Gel Robots

**Figure 1:**
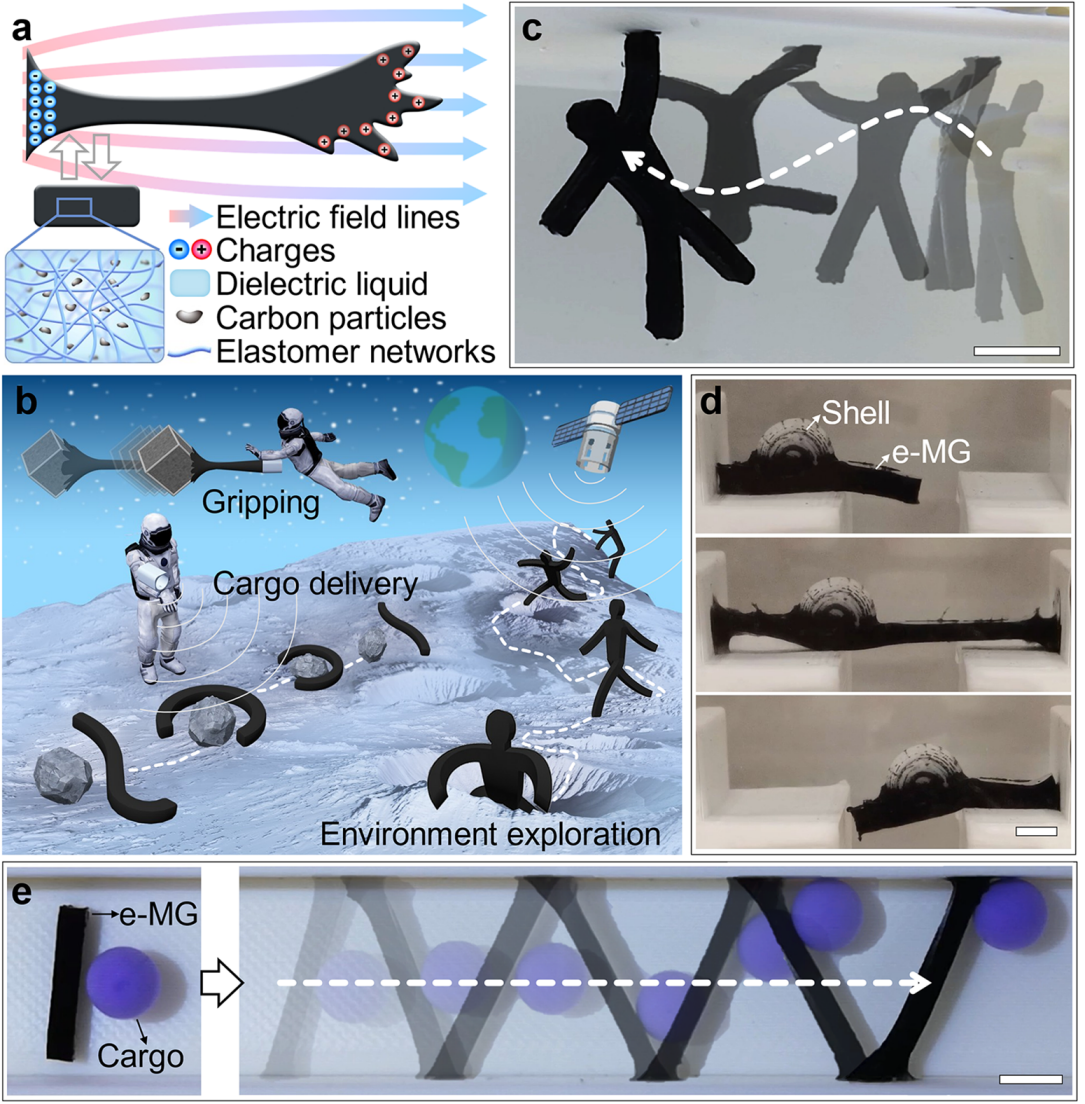
Demonstration of the deformability of e‐MG robots. a) Illustration of the e‐MG material structure and its principle of actuation under an electric field. b) Conceptual diagram showcasing the potential of e‐MG robots in space applications. c) An e‐MG gymnast swinging along a ceiling. d) An e‐MG snail jumping over a gap. e) An e‐MG robot delivering cargo through a channel. Demonstrations in (c–e) were performed in a dielectric liquid environment. Scale bars are 5 mm.

e‐MG robots are constructed from a soft polymer‐carbon composite gel and exhibit large‐scale electric field response due to charge separation and repulsion (**Figure** **1**a). They can achieve remote controllable and reversible deformation under the contactless stimuli of external and remote electric fields, analogous to magnetic robots under magnetic fields. Compared with magnetic counterparts (Table S1, Supporting Information), the complexity, size, and mass of the e‐MG robot control system are dramatically reduced while extending the morphing capability of the robots, because complex electric fields can be readily built by lightweight, compact, and arbitrarily shaped electrodes. In addition, magnetic soft robots often rely on pre‐programmed magnetization patterns,^[^ ^8^, ^9^, ^27^, ^28^, ^29^ ^]^ which require complex fabrication and limit actuation to fixed deformation responses. The e‐MG robots presented here do not need programming during fabrication and can undergo complex morphing by configuring electric fields. The characteristics of the material and actuation strategy of e‐MG open doors to a wide range of possibilities, including space applications. As shown in the conceptual diagram Figure 1b, we envision that e‐MG robots can play a significant role in accomplishing tasks during space missions, such as gripping, cargo delivery, and environment exploration by taking advantage of their extraordinary capabilities of remote morphing and locomotion.

The geometry of an e‐MG robot is tailorable depending on application scenarios. For example, in Figure 1c and Video S1 (Supporting Information), we demonstrated the robot as a jelly humanoid gymnast with an agile body and active limbs. The e‐MG gymnast can use its hands or feet to avoid falling while bending its body to move forward, swinging along the ceiling. The soft e‐MG robot can be combined with rigid components to generate more functional hybrid rigid‐soft robots. For example, In Figure 1d and Video S1 (Supporting Information), we demonstrated an e‐MG snail formed of a soft body and a rigid shell that can jump over a gap via first stretching and then contracting its body, mimicking the behavior of a snail in nature. The robot exhibits spreading deformation on the perpendicular wall for anchoring while stretching horizontally, which illustrates its capability for deformation. Untethered actuation endows significant flexibility to e‐MG robot locomotion, as presented in Figure 1e and Video S1 (Supporting Information). An e‐MG robot is able to move a ball through a narrow channel: the robot stretches its body and anchors to the wall at each step, capturing and pushing the cargo as it moves forward.

**Figure 2:**
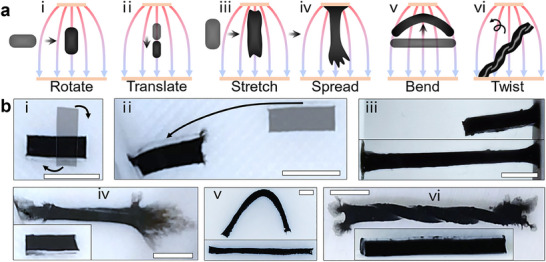
The diverse morphing patterns of e‐MG robots. a) Diagrams of electrical field driven actuation. (i) rotate, (ii) translate, (iii) stretch, (iv) spread, (v) bend, and (vi) twist. b) Examples of e‐MG actuation in a dielectric liquid environment. (i–vi) match (a). Scale bar are 5 mm.

As illustrated in **Figure** **2**, a unique feature of the e‐MG robots is their ability to perform diverse shape‐changing (stretching, spreading, and twisting), deformation (bending), and moving (rotating and translating) patterns. These behaviors depend on the polarity, intensity, and gradient of the applied electric fields. Rotation can be achieved when the direction of the applied electric field misaligns with the principal axis of the robot (Figure 2a(i)). When the robots are exposed to a field with low gradient and intensity, typically occurring when they are far from the electrodes, the force is insufficient to induce morphing. As a result, translation behavior is dominant (Figure 2a(ii)). The actuation force increases with the increase in intensity and gradient of the electric field, such as when the robot moves closer to the electrode. When the actuation force exceeds the mechanical stress, the robot undergoes stretching (Figure 2a(iii)). When the robot makes contact with the electrode, the most intense morphing occurs as spreading (Figure 2a(iv)). The reversible spreading behavior, where the surface‐to‐volume ratio increases significantly, is not observed in other soft robots. Morphology of the robots also affects the deformation behaviors, for example, bending is normally realized on robots with elongated bodies when they experience a non‐aligned electric field (Figure 2a(v)). Twisting (Figure 2a(vi)), which results from interaction with local friction forces, is most evident in an elongated robot body. This behavior occurs when one side of the robot is anchored or exposed to friction forces, and a non‐aligned electric field is applied. The abovementioned morphing and moving patterns can be achieved simultaneously (e.g., stretching with spreading) or sequentially (e.g., stretching after rotation), enabling more complicated deformations and thus more adaptability to the environment. Note that the generated electric field is also affected by the e‐MG robots due to their dielectric properties. As shown in Figure S1 (Supporting Information), the robot alters the uniformity of the electric field; for instance, the local field strength increases dramatically as the robot approaches the electrode. During morphing and locomotion, the geometry, size, and position of the robot affect the electric field, which in turn affects its actuation behavior. The manipulation of robots can be achieved by controlling the parameters of electrodes, such as voltage, stimulation signal, position of energizing electrodes, and electrode geometry, which will be discussed in the following sections.

### 2.2 Material Characterization and Actuation Principle

**Figure 3:**
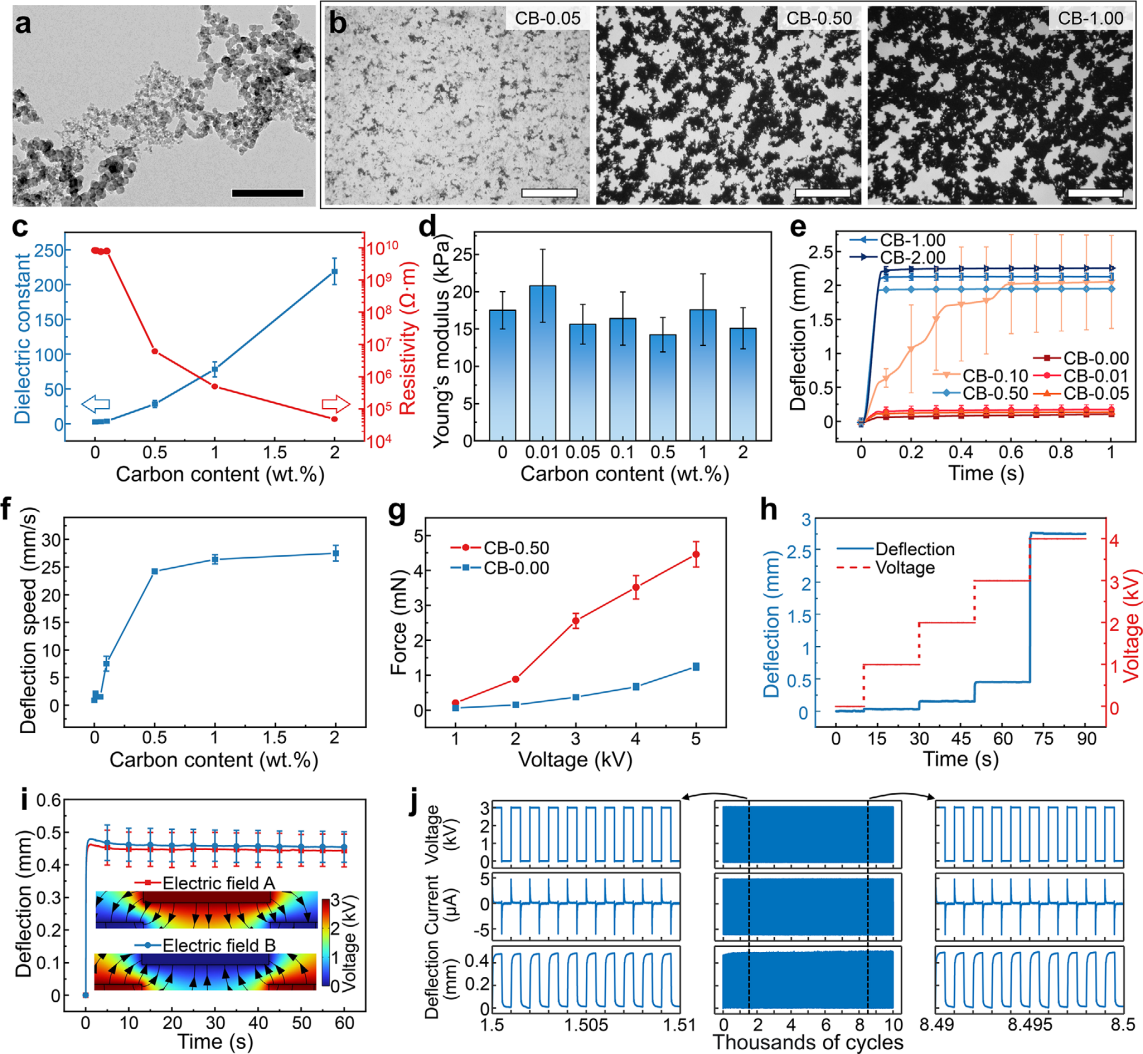
Material and actuation characterization. a) Transmission electron microscopy image of nano‐procrystalline carbon black powder. Scale bar is 0.5 µm. b) Optical microscopy images of e‐MG samples. Scale bars are 500 µm. Carbon black appears as dark in the images, and the elastomer matrix appears as a bright background. c) Dielectric constant and electrical resistivity. d) Young's modulus. e) Actuation deflection of different samples. f) Deflection speed. g) Actuation force of samples CB‐0.00 and CB‐0.50. h) Deflection response to a staircase voltage increase. i) Comparison of actuation under two reversed electric fields. Inserts are finite element simulations of the two experimental electric fields. Black arrows represent the electric field lines. j) Voltage, current, and deflection in a cyclic test, 10 thousand cycles at a frequency of 0.5 Hz. The results in (h–j) are presented for sample CB‐0.50. The characterizations were conducted in an air environment.

The unique capabilities of e‐MG matter arise from the interaction of its electrical and mechanical properties. As shown in Figure 1a, the e‐MG is constructed from three components: a soft elastomer matrix, dielectric liquid, and nanoparticulate paracrystalline carbon, each of which plays a crucial role. The elastomer matrix is responsible for shaping the geometry of the robot and restoring it after morphing. The dielectric liquid acts as a plasticizer, reducing the elastomer network density within the composite. This enhances the softness and deformability of the e‐MG, while also moderately affecting its dielectric constant and viscoelastic properties.^[^ ^30^ ^]^ The impact of dielectric liquid on the material properties of elastomer gels (without carbon additives) was investigated in our previous work.^[^ ^30^ ^]^ Nanoparticulate paracrystalline carbon, which exhibits high surface area and outstanding electrical conductivity, is distributed throughout the composite, providing free charges. The ability to form percolating networks at low concentrations, as well as its low density, makes carbon particles efficient for enhancing electrical properties with minimum impact on the mechanical properties of the composites. To explore the influence of carbon particles on e‐MG properties, different ratios of carbon ranging from 0.01 to 2 wt.% are introduced during e‐MG fabrication. The samples are labeled as CB‐0.00, CB‐0.01, CB‐0.05, CB‐0.10, CB‐0.50, CB‐1.00, and CB‐2.00, respectively, where the number represents the wt.% of carbon within the sample. The carbon particles are approximately spheroidal in shape, with the size of 6–144 nm (**Figure** **3**a). They tend to form clusters because of the strong van der Waals forces between nanoparticles.^[^ ^31^ ^]^ Optical microscopy images of e‐MG with different carbon ratios are shown in Figure 3b. Carbon particle clusters are randomly distributed in the elastomer matrix and, depending on the concentration, can connect with each other to form networks. The assembled carbon networks create conductive pathways that facilitate charge migration, impacting the material properties and actuation performance. The influence of carbon ratio on the dielectric constant and resistivity is shown in Figure 3c (see Figure S2a,b (Supporting Information) for frequency dependence of dielectric constant). The dielectric constant remains steady at around 3 when the carbon content is below 0.1%, but jumps dramatically to 28 and 219 when the carbon percentage is increased to 0.5% and 2%, respectively. The electrical resistivity curve reveals a similar tendency. The change of resistivity is minor when the carbon content is less than 0.1%, but increases significantly as the ratio exceeds 0.5%. The high electrically conductive carbon filler and the low electrically conductive elastomer matrix form a percolative composite. Electrical percolation occurs when the carbon content approaches the percolation threshold and conductive paths are constructed, resulting in abrupt changes in dielectric and electrical properties.^[^ ^32^ ^]^ The percolation threshold of this composite system is observed to be between 0.1 and 0.5 wt.%. The effect of carbon ratio on mechanical stress is also studied, as shown in strain‐stress curves (Figure S2c, Supporting Information) and Young's modulus (Figure 3d). The variation in the carbon ratio between 0% and 2% does not significantly affect the stress–strain properties, with a breaking elongation around 450%, a maximum stress of ≈29 kPa, and Young's modulus of 17 kPa. This is because the mechanical property of the composite mainly derives from the elastomer network,^[^ ^30^ ^]^ rather than the carbon.

The combination of dielectric elastomer and conductive nanoparticles creates a malleable gelatinous matter with dynamic plasticity and electric field controllability. Actuation deflection of samples with different carbon concentrations is shown in Figure 3e (see more results and discussion in Figure S2d and Section S1, Supporting Information). The actuation deformation of the sample without carbon (CB‐0.00) is induced by dielectrophoresis,^[^ ^30^ ^]^ with the deflection increasing from 0 to 1.3 mm in 35 s. The material is polarized instantly when it is exposed to an electric field. The dielectrophoretic force is generated due to the non‐uniformity of the electric field, pushing the material toward the area of higher field density. When a low concentration of carbon is added (less than 0.1%), percolating conductive networks have not yet formed in the material. In this case, the bulk dielectric constant and conductivity remain nearly constant (Figure 3c), resulting in minimal change in force, and consequently, the actuation deflection in Figure 3e remains almost unaffected. For samples containing more than 0.1% carbon, the e‐MG materials show increased conductivity. As a result, actuation behavior is influenced not only by the dielectrophoretic force but also by the electrostatic force, which redistributes charges within the material and enhances its deformation significantly. Therefore, samples CB‐0.50, CB‐1.00, and CB‐2.00 exhibit exceptional actuation performance due to the strong resultant force. Specifically, as shown in Figure 3f, the introduction of 0.5% carbon (CB‐0.50) increases the actuation speed by a factor of 27 compared to the sample without carbon (CB‐0.00) (24.2 vs 0.9 mm s^−1^). The difference in actuation speed between samples CB‐0.50, CB‐1.00, and CB‐2.00 is relatively small. This is attributed to the well‐established conductive network in these samples, which is sufficient for charge transport, making additional carbon content less impactful in further enhancing effective charge mobility. It is observed that carbon loading above 0.5% may lead to incomplete curing during fabrication; thus, the sample CB‐0.50 is chosen as the representative e‐MG material to perform all the tests and demonstrations in this article unless specified otherwise. The actuation force is illustrated in Figure 3g, showing that the sample CB‐0.50 (4.46 mN) is ≈3.5 times greater than the sample CB‐0.00 (1.25 mN) at 5 kV. The actuation deflection can be promoted by applying a higher voltage (Figure 3h; Figure S2e, Supporting Information), as increasing the voltage enhances the actuation force. In addition, both dielectrophoretic force and electrostatic force are independent of electric field polarity^[^ ^30^, ^33^ ^]^; thus, in Figure 3i, the difference between the two deflection curves is negligible when the electric field is reversed. Figure 3j presents the results of a cyclic test. The deflection behavior is almost unchanged over 10000 actuation cycles. The consistent actuation performance throughout the test illustrates the durability and reliability of the e‐MG, revealing its potential for long‐term applications without significant degradation in functionality. The rapid electroactive response of e‐MG under a step voltage and at various frequencies is demonstrated in Figure S3 and Video S2 (Supporting Information).

Moreover, the e‐MG is a good candidate material for space applications, as discussed in Section S2 (Supporting Information). The extremely low pressure in space allows for establishing electric fields with high intensity and thus enables strong e‐MG actuation, as the breakdown strength in space is greater than that of Earth's atmosphere (Figure S4a, Supporting Information). Experimental investigation (Figure S4b, Supporting Information) indicates that low pressure has negligible impacts on the e‐MG. Despite the challenging temperatures in space, the components that make up the e‐MG have separately been employed in space engineering and can withstand the operating temperature range of common spacecraft (Figure S4c, Supporting Information). The thermal stability investigation further illustrates the capacity of e‐MG to endure extreme conditions (Figure S4d–f, Supporting Information). In this article, we demonstrate the e‐MG in two media, air and dielectric liquid. Atmospheric air not only has almost the same dielectric constant as vacuum,^[^ ^34^ ^]^ but its breakdown voltage also acts as a suitable approximation for space (Figure S4a, Supporting Information). We conducted material and actuation characterization in air as a medium to simulate the dielectric conditions of space. The versatile capabilities of e‐MG robots can also be demonstrated in myriad dielectric media. Mineral oil, for example, can be used as the surrounding fluid based on the following considerations: 1) The density of mineral oil is close to, but smaller than, the e‐MG (0.84 vs 0.97 g cm^−3^). The buoyancy provided by the mineral oil reduces the net effect of gravity and surface friction, and the lubricating characteristics of mineral oil reduces resistance to robot movement, thus mimicking the microgravity environment of space; 2) The oil can prevent electric short‐circuit under high‐voltage operation, which enables the establishment of more complex and higher electric fields in a terrestrial test environment, demonstrating the actuation potential of e‐MG in the high breakdown strength environment of space; 3) The material properties of the e‐MG are unaffected by the mineral oil.

**Figure 4:**
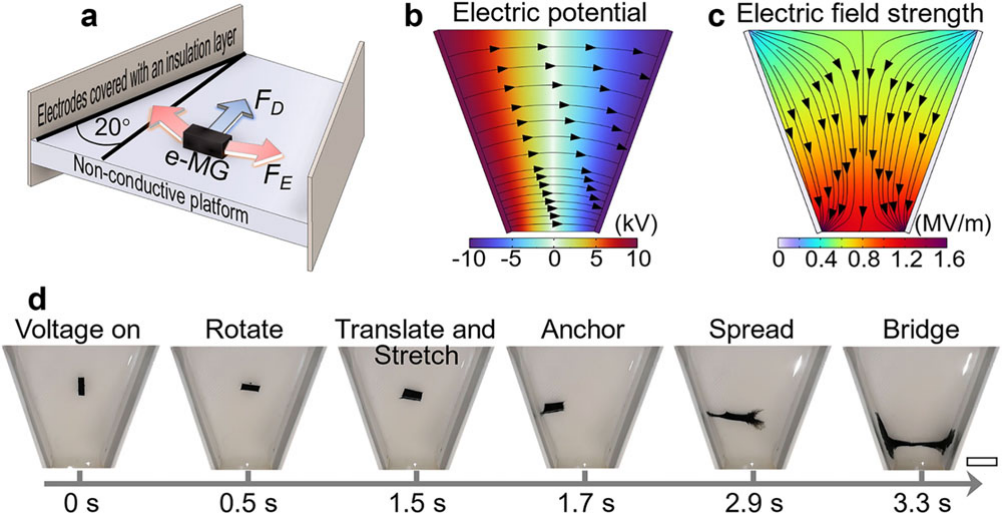
Electric field response mechanism. a) Diagram of the experiment setup. *F_E_* and *F_D_* represent electrostatic force and dielectrophoretic force, respectively. b) Finite element simulation of electric potential. Black arrows are electric field lines. c) Finite element simulation of electric field strength. Black arrows are electric field gradient lines. d) Response of an e‐MG robot in a dielectric liquid environment under direct voltage. The scale bar is 1 cm.

Next, we explore the underlying electro‐mechanical coupling principle of the e‐MG material in mineral oil to enable the future exploitation of e‐MG robots. To test the actuation in a non‐uniform electric field, we employ a platform with V‐shaped configured electrodes, as shown in **Figure** **4**a. The electrodes are covered with an insulation layer to prevent charge migration between them. Opposing potentials are applied to the electrodes to ensure the electric potential of the electrodes is distinguishable from the surrounding environment. For example, in Figure 4b–d, we apply 10 and −10 kV with respect to ground, to the left and right electrodes, respectively. The resulting electric field is distributed between the two electrodes (Figure 4b). The V‐shaped electrode configuration results in an inhomogeneous electric field (Figure 4c). When an e‐MG robot is exposed to this electric field, charge separation in the conductive networks, along with dipole and interfacial polarizations at the local scale, results in an unbalanced distribution of electric charges in the e‐MG material. Electrostatic forces and dielectrophoretic forces are thereby generated, leading to morphing of robots at both local and global scales.

The electric field simultaneously redistributes charges and induces polarization in the e‐MG material. The electrostatic force (*F_E_*) acting on the charges is given by:

(1) $$F_{E}=qE$$

where *q* is the charge in the material, and *E* is the local electric field at the charge location. The strength of *F_E_* depends on the magnitude of the electric field and the charge density in the material, and its direction aligns with the electric field, as illustrated by the electric field lines in Figure 4b. When the electric field is non‐uniform, the interaction between the electric field and a polarizable material leads to the dielectrophoretic force (*F_D_*). For a spherical particle with a radius of *R*, the force is expressed as:^[^ ^35^ ^]^

(2) $$F_{D}=2\pi R^{3}\varepsilon_{1}K\nabla E^{2}$$

(3) $$K=\frac{\varepsilon_{2}-\varepsilon_{1}}{\varepsilon_{2}+2\varepsilon_{1}}$$

where *ɛ*_1_ and *ɛ*_2_ are the permittivity of the surrounding medium and the actuated particle, respectively, *E* is the applied electrical field, *∇* is the gradient operator, and *K* is the Clausius–Mossotti factor. In this article, the permittivity of e‐MG is greater than the surrounding medium, i.e., *ɛ*_2_ > *ɛ*_1_; thus, *K* becomes positive, and the polarity of *F_D_* points toward the region of the higher electric field intensity. *F_D_* only appears in non‐uniform electric fields, and its magnitude increases with the strength of the field gradient. The polarity of *F_D_* follows the electric field gradient, as indicated by the black arrows in Figure 4c. The relative contribution of the two forces to the actuation of a specific e‐MG depends on the field gradient and field intensity. Since the conductive networks in the e‐MG material provide a high density of mobile charges, which redistribute across the e‐MG under a high‐intensity electric field, *F_E_* typically plays a dominant role in actuation (Section S3, Supporting Information).

Although in this article, we distinguish between *F_D_* and *F_E_* to describe the e‐MG's behavior based on the uniformity of electric fields, the two are inherently interconnected, as both originate from Maxwell stress.^[^ ^36^, ^37^, ^38^ ^]^ Models analyzing the force and the electro‐responsive actuation of e‐MG are developed and presented in Figure S5 and Section S4 (Supporting Information).

Figure 4d and Video S3 (Supporting Information) illustrate the reaction of an e‐MG robot to a direct voltage. As charges tend to accumulate at pointed ends of conductive materials, the robot is likely to show polarity in its longest axis during polarization. If this axis is not parallel with the electric field, the robot will rotate to realign and minimize potential energy, as is commonly observed in metallic wire alignment.^[^ ^39^, ^40^ ^]^ Thus, the robot rotates to lie parallel to the electric field within 0.5 s of the voltage being applied. The electrostatic forces attract the robot to the two electrodes, acting in opposite directions on the two ends of the robot, causing it to stretch. Simultaneously, the dielectrophoretic force pulls the robot toward the lower corner of the V‐structure where the field strength is greatest. Once the robot contacts the electrode, polarization is enhanced, and more charges are accumulated at the ends of the robot, resulting in a further increase in mechanical response to the electric field. First, one side of the robot is anchored on the electrode by the strong attraction force (1.7 s), and then the other side is pulled toward the counter electrode. At the same time, the increased charge accumulation at the free end of the robot generates large local repulsive forces that act to pull the end of the robot apart, leading to the unique spreading behavior (2.9 s). As demonstrated in Figure S6 and Video S4 (Supporting Information), the spreading behavior can result in an extremely large and rapid deformation, reaching 286% strain with a strain rate up to 500% s^−1^. This behavior is analogous to the dynamic responses of slime mold to environmental stimuli, spreading out in a branching and network‐like pattern to maximize its reach. Finally, the robot bridges the gap between the two electrodes (3.3 s). To illustrate the effect of different actuation forces, a 100 Hz alternating voltage was applied to the system (Figure S7 and Video S3, Supporting Information), where the electrostatic forces are weakened by the constantly changing voltage polarity. In comparison, the rotation (1 s) and translation (10 s) are delayed, and the robot moves toward the lower corner from the beginning. The dielectrophoretic stretching and spreading phenomena still occur in the AC case when the robot approaches the high‐intensity field at the corner. Locomotion and deformation of the robot in the AC field also finish with electrode bridging (12.2 s). The faster and stronger response in the DC case reveals that the contribution of the electrostatic force is important. Therefore, we employ direct voltage in e‐MG robot demonstrations, except as otherwise noted.

### 2.3 Manipulation with Non‐Planar Electrodes

**Figure 5:**
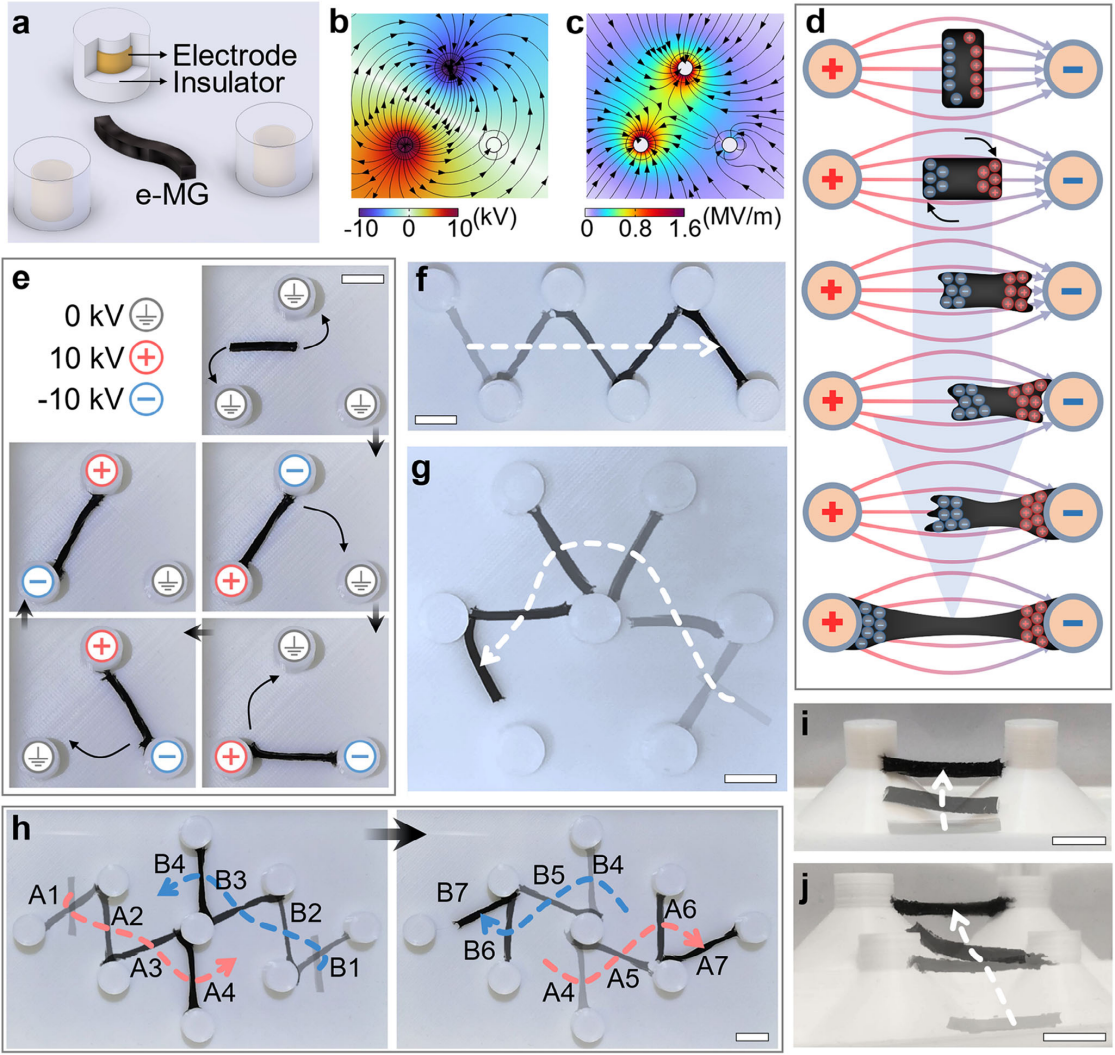
Manipulation with cylindrical electrodes. a) Diagram of cylindrical electrode platform. b) Finite element simulation of electric potential. Black arrows are simulated electric field lines. c) Finite element simulation of electric field strength. Black arrows are simulated electric field gradient lines. d) Diagram of e‐MG robot reaction when placed between two electrodes. e) Locomotion demonstration on a three‐electrode platform. f) Straight locomotion. g) Circular locomotion. h) Simultaneous and independent manipulation of two e‐MG robots, labeled A and B for distinction. The numbers indicate the sequence of locomotion steps. i) Locomotion on a conical surface. j) Locomotion on two pairs of stepped conical surfaces. Demonstrations were performed in a dielectric liquid environment. Scale bars are 1 cm.

The net actuation force acting on the robot can be maximized when the electrostatic force and dielectrophoretic force have the same polarity. Hence, we explored a point‐based electrode control system, as illustrated in **Figure** **5**a. Cylindrical electrodes are distributed uniformly on a flat platform, where adjacent electrodes maintain a consistent distance. Electrodes are electrically insulated to prevent charge migration among electrodes. We applied opposing potentials to two of the electrodes (e.g., 10 and −10 kV) and grounded all other electrodes. In this electrode configuration, both the electric field (Figure 5b) and field strength gradient (Figure 5c) are radial, aligned, and in the same direction in the vicinity of energized electrodes.

The diagram in Figure 5d shows the response of the e‐MG robot positioned between two cylindrical electrodes. After turning on the voltage, charge separation and polarization occur in the e‐MG, causing it to rotate to orientate to the electric field. It then translates toward one side due to the unbalanced force between the electrodes. Polarization increases when the robot approaches the first electrode. This results in anchoring on one side of the robot and fiercer morphing on the other side. The robot is strongly pulled to the other electrode through stretching and spreading until it bridges the two electrodes.

Optionally, we can include an intermediate state where voltage is only applied to one electrode and the others are grounded. In this case, both the electric potential and field gradient are concentrated around the energized electrode (Figure S8, Supporting Information), helping to anchor the robot.

A demonstration of more complex manipulation is conducted by three cylindrical electrodes, as depicted in Figure 5e and Video S5 (Supporting Information). The robot attempts to bridge the two nearest energized electrodes. If a different electrode is then energized, the robot will move to the new bridging position. Continuous locomotion is enabled by shifting the electric field around the robot, which can be achieved by controlling the voltage between the electrodes. If more electrodes are employed, the range of movements can be extended, enabling more flexible and sophisticated navigation. For example (Video S5, Supporting Information), an e‐MG robot can move along a figure‐of‐eight path (Figure S9, Supporting Information), a straight route (Figure 5f), and a curved route (Figure 5g). A twisting behavior can also be generated during locomotion, especially when one side of the robot is anchored. Local friction forces can be generated at or near the electrodes, and subsequent translational forces cause the material to twist about its long axis.

This simple electric field control strategy allows simultaneous and independent manipulation of multiple robots. In Figure 5h and Video S5 (Supporting Information), two robots move across an array of electrodes through different paths in opposite directions. Although the two robots can come close to each other, they exhibit independent movement. This is because two localized electric fields can be created and manipulated on different sides of one electrode at the same time. Notably, the individual manipulation of diverse neighboring robots is typically not possible in magnetic responsive matter, as it is difficult to build a localized magnetic field at such close distances, especially using electromagnetic actuation systems.^[^ ^27^, ^41^, ^42^, ^43^ ^]^

We extend e‐MG robot locomotion and manipulation to 3D conical surface as shown in Figure S10a–c (Supporting Information). When voltage is applied to the electrodes, the e‐MG robot rolls up the conical surface against gravity to reach the peak (Figure 5i; Video S6, Supporting Information). The distribution of the electric field (Figure S10d,e, Supporting Information) results in the actuation force pointing toward the electrode. The geometry of the cone causes a component of the force to apply parallel to the conical surface, driving the robot along the surface (Figure S10f, Supporting Information). The rolling behavior is the result of the interplay between driving force and friction. By using the same strategy, the robot can roll up two stepped sets of conical surfaces (Figure 5j; Video S6, Supporting Information), illustrating the ability of e‐MG robots to move in 3D space.

### 2.4 Manipulation with Planar Electrodes

**Figure 6:**
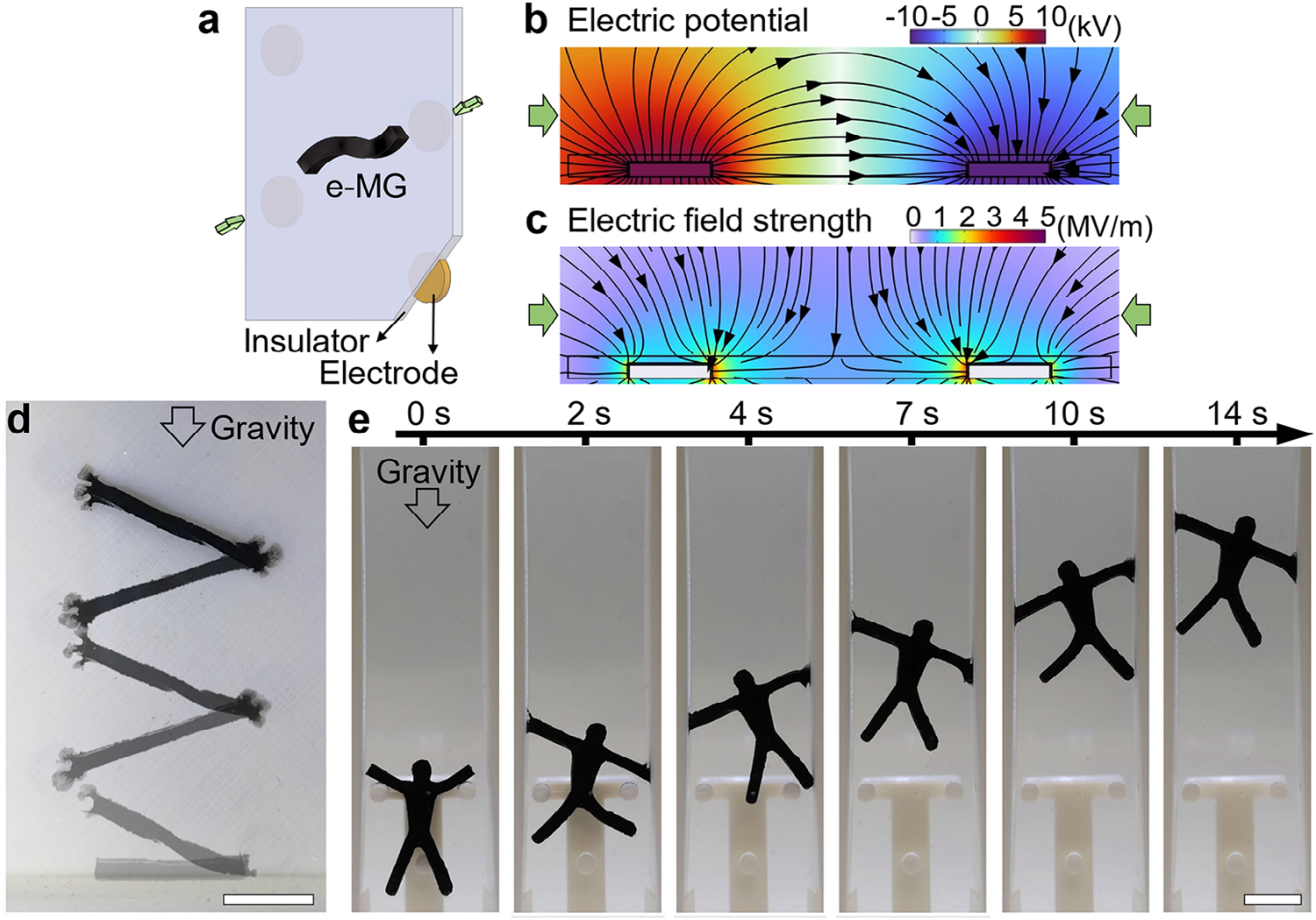
Manipulation with planar electrodes. a) Diagram of the planar electrode platform. b) Finite element simulation of electric potential. Black arrows are simulated electric field lines. c) Finite element simulation of electric field strength. Black arrows are simulated electric field gradient lines. Green arrows in (b, c) align with those in (a). d) Demonstration of an e‐MG robot moving up a vertical flat surface against gravity. e) Demonstration of an e‐MG gymnast moving up a vertical channel. Demonstrations were performed in a dielectric liquid environment. Scale bars are 1 cm.

The electrical manipulation platform is not limited to cylindrical electrodes but can be of almost any structure. As shown in **Figure** **6**a, electrodes can be attached to the back of a flat surface. When we apply 10 kV to one electrode and −10 kV to the adjacent electrode, the electric field through a cross‐section of the structure (indicated by green arrows in Figure 6a) is shown in Figure 6b,c. Both electric field lines and field gradient lines converge at the electrodes, which suggests the strongest actuation force and anchoring point. Therefore, the e‐MG robot tends to approach and bridge these areas. When the driving force is greater than gravity, the robot can climb up a vertical wall, as illustrated in Figure 6d and Video S7 (Supporting Information). After each step, the robot bridges the electrodes and undergoes dramatic morphing at its ends. The generation of finger‐like branches indicates that the robot is anchored to the wall, which prevents falling. When the voltage becomes zero, the “fingers” retract back into the body, and the robot releases from the wall. Similarly, a strip e‐MG robot can deliver cargo in a horizontal channel (Figure 1e; Video S1, Supporting Information). As demonstrated in Figure 6e and Video S8 (Supporting Information), an e‐MG gymnast can climb up a vertical channel with a speed of 3.1 mm s^−1^. The electrodes located on the upper sides of the robot are energized step by step to enable upward climbing. Here, the “hands” are positioned closest to the active electrodes, where the electric field is stronger. Thus, the “hands” are more significantly influenced by the electric field and act to bridge the channel. Climbing locomotion is achieved through a gripping and releasing motion of the “hands” against the channel walls. The swinging upside‐down gymnast locomotion shown in Figure 1c and Video S1 (Supporting Information) is realized by exploiting the same strategy.

### 2.5 Demonstrations of Morphing, Gripping, and Manipulation in Air

**Figure 7:**
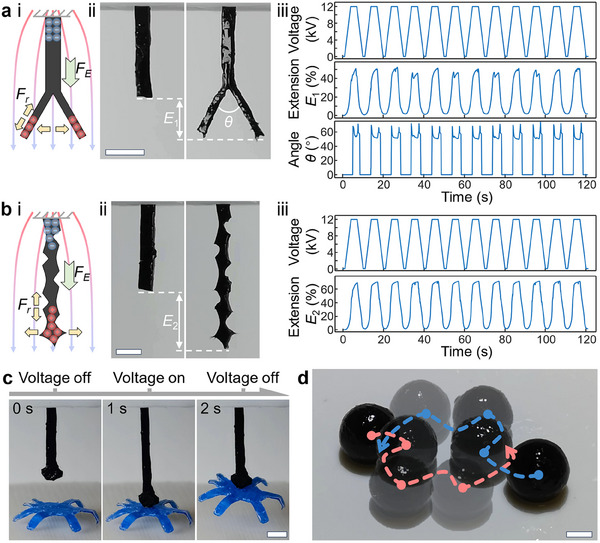
Demonstrations of morphing, gripping, and manipulation in the air. a) Diagram (i), photographs (ii), and experimental results (iii) of a structured e‐MG strip morphing with two branches. *F_E_* and *F_r_* in (i) are the electrostatic force from the electrode and the repellent force between like charges, respectively. b) Diagram (i), photographs (ii), and experimental results (iii) of a morphing kirigami e‐MG. c) A frog tongue‐inspired e‐MG gripper. d) Demonstration of the simultaneous manipulation of two e‐MG spherical robots. Scale bars are 2 mm.

To demonstrate that similar actuation behavior is achievable in different media, we showcase morphing, gripping, and manipulation in air. Actuation performance can be influenced by the media, as presented in Figure S11 and Section S5 (Supporting Information). The electric field‐driven strategy endows the e‐MG with exceptional morphing capabilities by pushing like charges to the opposite ends of the e‐MG (**Figure** **7**a(i)). The e‐MG then exhibits deformation under the influence of the electrostatic force induced by the electrodes and the repellent force between like charges. As demonstrated in Figure 7a and Video S9 (Supporting Information), the whole e‐MG strip is stretched 50%, and the branches are separated at an angle (≈60°) due to repulsion. The electric field‐induced morphing is more obvious in thinner areas of the e‐MG, as charges are more likely to accumulate in sharper points (local field enhancement), and there is less mechanical stiffness to overcome. Leveraging structural design can further enhance actuation extension. As shown in Figure 7b and Video S9 (Supporting Information), a kirigami e‐MG strip smoothly elongates under the applied ramp‐increasing voltage, reaching a maximum extension of 71% before the voltage starts to decrease. The controllable elongating behavior can be exploited to develop grippers (Figure 7c; Video S9, Supporting Information), which mimic the tongues of frogs or chameleons during hunting. When the elongated e‐MG gripper contacts the object, the target is captured by taking advantage of the adhesive nature of the e‐MG. With the voltage switched off, the e‐MG recovers to the original length, and thus the object is lifted in the air. The gripping process takes less than 2 s. The locomotion and manipulation of the e‐MG can be achieved by configuring electrodes under a platform. When near electrodes are energized, the adjacent e‐MG is polarized, causing it to be attracted to, and roll into, the region of the strong electric field. In this strategy, the electric field is concentrated between the two energized electrodes, providing opportunities to establish multiple localized electric fields at a close distance. Therefore, multiple neighboring e‐MG robots can be manipulated independently. As presented in Figure 7d and Video S9 (Supporting Information), two e‐MG spherical robots in close proximity are able to approach, turn, and diverge independently.

## 3 Conclusion and discussion

The soft morphing matter robots demonstrated in this article show extremely high morphological adaptability and complex multimodal electric field response. They can undergo large, controlled body deformation and show complex locomotion on different terrains, including 3D surfaces, walls, ceilings, and channels. The relationships between electric field configurations and morphing patterns were explained. Actuation characterization combined with material investigation reveals the dielectrophoretic force and electrostatic force behind the electric field navigation. We demonstrated control of robots using cylindrical electrodes and flat surfaces with embedded planar electrodes, providing practical platforms for future development. Actuation behaviors in various media, including air and dielectric liquid, were presented, and the potential for actuation in space was discussed, which highlights the versatility of e‐MG robots for diverse application environments.

Unlike dielectric elastomer actuators (Figure S12 and Section S6, Supporting Information), which utilize internal electric fields for actuation, the electric field‐driven actuation introduced here is a remote‐control strategy, with electric fields constructed externally around the robots. This strategy provides solutions to robot manipulation in situations where other fields, such as light, heat, acoustic, and magnetic, are impractical, uneconomical, or impossible. For instance, in environments where photonic and magnetic fields cannot pass through the surrounding medium, or in systems necessitating a light weight, low cost, and simple robot with arbitrary complex deformability. Magnetic field responsive soft robots have been widely investigated and demonstrate high deformability and controllability. However, their control systems are usually cumbersome, and it is difficult to confine magnetic fields to a specific area for precise control. e‐MG robots are not only able to achieve similar morphing and locomotion patterns (Table S1, Supporting Information), but electric field‐driven actuation also allows for more localized and independent manipulation by applying different voltages to specific regions, as demonstrated in Figures 5h and 7d. Besides, shielding of magnetic fields to protect electronic devices typically requires bulky and heavy ferromagnetic materials.^[^ ^44^, ^45^ ^]^ In contrast, electric fields can be more easily shielded and controlled with lightweight and flexible materials.^[^ ^46^ ^]^

The scalability of e‐MG robots is demonstrated in Figure S13 and Video S10 (Supporting Information). Although the volumes of the two robots are over 4000 times different, they exhibited the same actuation modes, from rotating, stretching, anchoring, spreading, to bridging. The capability to scale across sizes increases their adaptability, enabling a wider range of industrial and robotics applications. Particularly, future miniaturized e‐MG robots could perform precision tasks such as assembling microelectronics or passing through narrow pathways for in situ monitoring. Moreover, as electrodes are the sources of electric field for manipulation, the large design space of electrodes endows e‐MG robots with myriad possibilities. The advantage of electrodes lies in their significant flexibility for customization across various aspects, from dimension and morphology to material phase; electrodes can be solid (e.g., machine cut foil), liquid (including liquid metal), or colloid. While most of the electrode configurations presented in this article are designed on a flat surface, thereby limiting locomotion to a planar area, it is anticipated that more sophisticated locomotion and manipulation of e‐MG robots, such as multi‐robot coordination, can be achieved by employing more complex morphologies and configurations of electrodes. Utilizing mobile electrodes to generate a dynamically adjustable electric field in real time offers an alternative strategy, for example, robotic arm‐assisted electrodes for intricate navigation or drone‐based electrodes for exploration missions. The e‐MG robots can also be utilized for picking up and transporting loads by leveraging their stickiness (Figure 7c), embedding objects within them (Figure 1d), or morphing to wrap around the items. Although their soft structure limits their capacity to carry heavy loads, they show great potential for handling delicate objects. Improving material properties to make e‐MG more sensitive to electric fields is an effective strategy to enhance remote controllability, offering opportunities to reduce applied voltage or to deploy electrodes further from robots without sacrificing actuation performance. The wireless electric field‐driven morphing matter robots presented here make a significant step beyond current electroactive and soft robots, providing a paradigm for the development of next‐generation smart robots.

## 4 Experimental Section

### Electro‐Morphing Gel Fabrication

The electro‐morphing gel (e‐MG) was constructed from three components: a silicone rubber elastomer (Ecoflex Gel, Smooth‐On), dielectric liquid (silicone oil, Sigma–Aldrich, the viscosity is 50 cSt at 25 °C), and carbon black (Cabot, VXC72R, particle size ≈6–144 nm). The three components were mixed by a mixing machine (ARE‐250, THINKY, Japan) at a speed of 2000 rpm for 4 min. The mixture was poured into 3D‐printed polyvinyl alcohol (PVA) molds (Ultimaker S5, Ultimaker, Netherlands) and then degassed in a vacuum chamber for 10 min. The e‐MG samples were cured at an ambient temperature for 4 h. Due to the water‐soluble feature of PVA, the cured sample was removed safely by dissolving the molds in water.

The Ecoflex Gel precursor contains two parts, mixed in the ratio 1:1 by weight. The mixing ratio between Ecoflex Gel precursor and silicone oil was fixed at 3:7 by weight, while the percentage of carbon black within the sample ranged from 0.01 to 2 wt.%. According to different carbon black ratios, the corresponding samples were labeled as CB‐0.01, CB‐0.05, CB‐0.10, CB‐0.50, CB‐1.00, and CB‐2.00, respectively, where the number represents the wt.% of carbon black. Samples without any carbon black were also fabricated for comparison and labeled as CB‐0.00.

### Microscopy Measurements

The transmission electron microscopy image of the carbon powder was obtained using a transmission electron microscope (FEI Tecnai 12). Optical microscopy images of the e‐MG samples were obtained on a digital microscope (KH‐7700, Hirox, Japan).

### Dielectric Constant

The dielectric constant was obtained using the parallel plate capacitance method. The wafer‐shaped samples (25 mm diameter, 1 mm thickness) were sandwiched by a pair of parallel cylinder electrodes (20 mm diameter). A precision LCR meter (E4980AL, Keysight, USA) was connected to the electrodes and measured the electrical capacitance (*C_p_*) at ambient temperature. The dielectric constant (*ɛ’*) was calculated from the equation:

(4) $$\varepsilon^{\prime }=\frac{C_{p}\times T}{\varepsilon_{0}\times S}$$

where *T* is the thickness of samples, *ɛ_0_* is the vacuum dielectric constant (8.85 × 10^−12^ F m^−1^), and *S* is the area of the electrode. Each sample was measured three times. Frequency dependence (from 1 to 100 kHz) of dielectric constant is shown in Figure S2a,b (Supporting Information). Figure 3c shows the dielectric constant at 10 kHz.

### Resistivity Measurement

Samples were prepared in wafer shape with a diameter of 25 mm and a thickness of 1.5 mm, and sandwiched by two cylinder electrodes with a diameter of 20 mm. A constant voltage (*U*) was applied to the electrodes while the current (*I*) was measured. The resistivity (*ρ*) was calculated by:

(5) $$\rho =\frac{U\times A}{I\times T}\;$$

where *A* and *T* represent the surface area and thickness of samples, respectively. Each sample was measured three times. There is a significant difference in the resistivity values among samples with different carbon content. If a constant voltage is applied to all the samples for current measurement, the resulting values will show significant variation. Considering measurement instruments have a limited accuracy range, different voltages were applied for specific samples to ensure the accuracy of the current measurement. 1 kV was applied to the samples CB‐0.00, CB‐0.01, CB‐0.05, CB‐0.10, and 50 V to the samples CB‐0.50, CB‐1.00 via a high voltage amplifier (5HVA24‐BP1, UltraVolt, USA). 6 V was applied to the most conductive sample CB‐2.00 via a potentiostat (HA‐151B, Hokuto Denko, Japan).

### Tensile Test

The two ends of the strip sample were securely fixed by two clamps, with the initial gauge dimensions of 10 (*L_0_*, length) × 15 (width) × 2 (thickness) mm. Samples were uniaxially stretched along the length direction by a tensile test machine (HY‐UT‐5 PC, Dong Guan Hongjin test instrument co., Ltd, China) with a 10 N load cell at a speed of 50 mm min^−1^ until break. A camera recorded the front view of the stretching process. The uniaxial force (*F*) was obtained from the tensile test machine, while the length (*L*) of the gauge section was extracted from the recorded video using MATLAB. Engineering strain was defined as ((*L*−*L_0_*)/*L_0_*) *×* 100%. It is assumed that the volume of the gauge section (*V*) remained constant during the stretching process, with the value of 10 × 15 × 2 = 300 mm^3^. The relationship between volume, length, width (*W*), and thickness (*T*) of the gauge section could be described as *V* = *L* × *W* × *T*; thus, *W* × *T* = *V*/*L*. Stress was defined as *F*/(*W × T*) = (*F × L*)/*V*. Three specimens were tested for each sample and presented the average values on the stress–strain curves. The Young's modulus was determined using the secant modulus at 20% strain from the stress–strain curves.

### Actuation Deflection and Force Measurement

The actuation deflection measurement setup is shown in Figure S14a–c (Supporting Information). The e‐MG membrane (1.5 mm thickness) was placed on an annular electrode (inner diameter 15 mm), and a cylinder electrode (15 mm diameter) was fixed above e‐MG, leaving a gap between the two. The actuation tests were carried out in air. The two electrodes were covered with an insulation layer (PVC tape) to prevent charge injection from the electrodes to the e‐MG. A laser displacement meter (LK‐G152 laser head, Keyence, Japan) was employed to measure the deflection of the bottom center of the e‐MG membrane. Voltage was applied through a computer‐controlled data acquisition system (NI USB‐6343, National Instruments, USA) and a high voltage amplifier (5HVA24‐BP1, UltraVolt, USA). The deflection values were recorded with respect to time, and each sample was repeated 5 times. The average results and standard deviation are shown in Figure 3e and Figure S2d,e (Supporting Information). The actuation speed shown in Figure 3f was extracted from the initial slope from 0 to 0.08 s of the time‐deflection curve. Each curve in Figure 3i was the average result of 14 repeated tests based on 6 different specimens of CB‐0.50. Results in Figure S2e (Supporting Information) show 5 repeated measurements for each voltage condition.

The half‐section profile of the e‐MG membrane curves downward slightly due to the softness of the material and the influence of gravity. The slagging displacement was ≈0.7–1.1 mm, as indicated by the red dashed lines and arrows in Figure S14c (Supporting Information). During actuation, the e‐MG membrane exhibits out‐of‐plane deformation toward the top electrode (Figure S14d, Supporting Information). As the top electrode was the limit of deflection, increasing the distance between the two electrodes (*d*) can enlarge the maximum deflection, although this compromises the actuation performance. For demonstrations, *d* = 2.5 mm was used in Figure 3e–g and Figure S2d (Supporting Information), but *d* = 3 mm in Figure 3h–j, Figures S2e and S3, and Video S2 (Supporting Information).

The actuation force measurement method is shown in Figure S14e (Supporting Information). The gel membrane was connected to a 50 mN force sensor (LVS‐5GA, KYOWA, Japan) through a nylon connector (non‐conductive and rigid). Isometric force measurements were performed while the membrane was kept in a flat profile. Each measurement was repeated 4 times.

### Finite Element Analysis

The simulation of the electric field was studied using the software COMSOL Multiphysics 6.1 (Electrostatic interface). The dimensions and materials in the simulation models were defined according to experimental conditions. Relative permittivity of different materials, including mineral oil, polylactic acid (PLA), polyvinyl chloride (PVC), and copper, were defined as 2.4,^[^ ^47^ ^]^ 2.8,^[^ ^48^ ^]^ 4.6,^[^ ^49^ ^]^ and 1, respectively.

### Manipulation Demonstration Methods

The e‐MG robot manipulation platforms were 3D‐printed (Mega S, Anycubic, China) from PLA filament. Cylindrical electrodes were made by wrapping one layer of copper foil (AT525, Advance, 0.035 mm thickness) onto the surface of the PLA cylinders. Planar electrodes were round brass wafers (6 mm diameter, 1 mm thickness). All the electrodes were covered with one layer of PVC tape (AT7, Advance, 0.13 mm thickness) and enclosed in a designed structure for electrical insulation. The dimensions of the cylindrical electrode setup (Figure 5), vertical wall setup (Figures 1c and 6a–d), and channel setup (Figures 1e and 6e) are shown in Figure S15 (Supporting Information).

The voltage of electrodes was controlled via a system that contains high voltage amplifiers (10HVA24‐BP1, UltraVolt, USA), high voltage relays (H12‐1A69, Meder), and data acquisition devices (NI USB‐6343, National Instruments, USA). The dielectric liquid for the demonstration environment is mineral oil (Sigma–Aldrich, 330779).

For the demonstrations in Figure 7a–c, the setup in Figure S16a (Supporting Information) was employed, which was constructed by two perpendicular planar electrodes. Applying high voltage in air can caused an electrical discharge when the electric field strength exceeded the dielectric strength of air. To avoid the electrical breakdown, the electrodes were covered with an insulation layer (PVC tape) and spaced at a distance from each other. The e‐MG strip, lightly wetted with mineral oil to reduce surface stickiness and enable opening of the structures, was fixed on the top electrode with the main body hanging in air. The top and bottom electrodes were applied with positive and negative voltage, respectively, with the same magnitude. The voltage results in Figure 7a(iii),b(iii) were the total electrical potential between the two electrodes. An object may become polarized when placed in an electric field. To minimize the influence of electrostatics on the target object, in Figure 7c, a 500 Hz AC voltage (± 8 kV) was applied to the electrodes. The demonstrations in Figure 7d were performed by using the setup in Figure S16b (Supporting Information). Opposite voltages (± 8 kV) with a 2 Hz frequency (on and off) were applied to two electrodes and lightly lubricated the platform with mineral oil to reduce the rolling resistance.

## Conflict of Interest

The authors declare no conflict of interest.

## Supporting information



Supporting Information

Supplemental Video 1

Supplemental Video 2

Supplemental Video 3

Supplemental Video 4

Supplemental Video 5

Supplemental Video 6

Supplemental Video 7

Supplemental Video 8

Supplemental Video 9

Supplemental Video 10

## Data Availability

The data that support the findings of this study are openly available in the University of Bristol Research Data Repository at https://doi.org/10.5523/bris.v9kmwppw3lk12qnjpepnz3xxz, reference number 50.
