# Supplementary material for: Electric Field Driven Soft Morphing Matter
Source: Adv Mater. 2025 Jun 12;37(41):2419077. doi: 10.1002/adma.202419077 (PMC12531759; doi:10.1002/adma.202419077)
Supplement: Supplementary file 1 — Supporting Information [file ADMA-37-2419077-s011.pdf]

ADVANCED MATERIALS

Supporting Information

for *Adv. Mater.*, DOI 10.1002/adma.202419077

Electric Field Driven Soft Morphing Matter

Ciqun Xu, Charl F. J. Faul, Majid Taghavi and Jonathan Rossiter**

Supporting Information

Electric field driven soft morphing matter

*Ciqun Xu, Charl F. J. Faul, Majid Taghavi *, Jonathan Rossiter **

Contents

Supplementary Text

Section 1. Actuation deflection of samples with low carbon contents 2
Section 2. Discussion of e-MG space applications 3
Section 3. The contribution of forces in actuation 6
Section 4. Models of forces and e-MG robot actuation 7
Section 5. Comparison of actuation in different media 13
Section 6. Comparison with dielectric elastomer actuators 14

Supplementary tables and figures

Table S1. Characteristics of e-MG and magnetic robot systems 15
Figure S1. Finite element simulation 16
Figure S2. Material and actuation characterization 17
Figure S3. Demonstrations of e-MG actuation in air environment 18
Figure S4. Voltage, pressure, and temperature considerations for space applications 19
Figure S5. Models of forces and e-MG robot actuation.. 20
Figure S6. Spreading demonstration of e-MG 21
Figure S7. Behaviors of e-MG in V-shape configured electrodes under a 100 Hz alternating voltage 22
Figure S8. Finite element simulation of electric field when only one cylindrical electrode is energized 23
Figure S9. Manipulation of an e-MG robot through a figure of eight path 24
Figure S10. Conical platforms 25
Figure S11. Comparison of actuation elongation and stored force between air and mineral oil actuation environments 26
Figure S12. Comparison with a dielectric elastomer actuator 27
Figure S13. Demonstration of scalability 28
Figure S14. Diagram of actuation deflection and force measurement setup 29
Figure S15. Diagram and size of the electrode configurations used 30
Figure S16. Setup diagrams of demonstrations in air 31
List of Supplemental Videos.. 32
References 33

Supplementary Text

Section 1. Actuation deflection of samples with low carbon contents

In actuation deflection characterization, samples with more than 0.1 % carbon can reach the deflection peak in 0.1 s, while samples with low carbon ratios (0 - 0.05 %) exhibit small deflection within the 1st second (Figure 3e). The actuation deflection after 1 s is shown in Supporting Information Figure S2d. It is interesting to note that the deflection of sample CB-0.00 reaches its peak in 35 s, whereas the deflection of samples CB-0.01 and CB-0.05 remain almost unchanged. The weak actuation of samples CB-0.01 and CB-0.05 could be attributed to the influence of carbon fillers on the electrical and mechanical properties of the material, such as the increased dielectric loss and polymer chain disruption [1-4], although this effect is not noticeable on a macro scale. The deformation is less than 2 % strain during the deflection tests, and we illustrate the stress-strain curves of this region in Supporting Information Figure S2f. Compared to sample CB-0.00, samples CB-0.01 and CB-0.05 require larger strain to achieve the same stress, which reveals the impact of carbon filler on mechanical properties in low-strain regions. A detailed exploration of this effect will be part of our future work.

Section 2. Discussion of e-MG space applications

Paschen's law is used to explain the relationship between breakdown voltage (V_b) and atmospheric pressure, which can be described by the equation [5-7]:

$$V_b = \frac{Bpd}{ln\left(\frac{A}{ln\left(1+\frac{1}{\gamma}\right)}\right)+ln\,(pd)} \quad \text{(S1)}$$

Where B is a constant related with the excitation and ionization energies, p is the gas pressure, d is the distance between electrodes, A is the saturation ionization in the gas, γ is secondary ionization coefficient. The values of B, A, and γ vary with experimental conditions [8-9]. For air study, we apply B = 273.75 V·(mbar·cm)$^{-1}$, A = 11.25 (mbar·cm)$^{-1}$, and γ = 0.01 [6, 9]. The Paschen curve for air according to Equation S1 is plotted in Supporting Information Figure S4a. The value of breakdown voltage tends to decrease to a minimum as pd is reduced, then shows a sharp increase when pd is decreased below this point. The breakdown tendency around this minimum is also observed experimentally [7-9]. We also note that atmospheric pressure is a function of altitude, decreasing as altitude increases [10-11]. To understand the influence of altitude on breakdown voltage, we assume that d = 1 cm and use pressure value to label altitude on Paschen's curve (Figure S4a). When the altitude is higher than 60 km, the breakdown voltage can reach (point B) and exceed the value at sea level (point A), bridging the region of low breakdown voltage. Therefore, the actuation test in our air environment (approximately sea level) can simulate the breakdown voltage condition at around 60 km altitude. The low earth orbit is typically between 160 and 2,000 km above Earth with the pressure between 10^{-11} and 10^{-13} mbar [12-13]. The lunar surface has similar pressure as low earth orbit [14]. Since the pressure in space environments is significantly lower than that of our experimental vacuum conditions, space environments exhibit a higher breakdown strength according to Paschen's law, allowing for potentially higher voltage operation and thus stronger e-MG actuation.

To reveal the effect of low pressure on the e-MG, e-MG samples were placed in a vacuum chamber (5 mbar), while control samples stayed in ambient pressure (1013 mbar). The mass was measured after at multiple time points and the results are presented in Figure S4b. The mass change of the samples is less than 1.5 % after 24 hours. Subsequently, an actuation elongation test was conducted for all the samples, as shown in the insert of Figure S4b. Two insulated electrodes were positioned 35 mm apart in air, with a strip e-MG sample (0.5 × 1 × 10 mm)

attached to the top electrode. When voltage was applied to the electrodes, the sample extended towards the bottom electrode. The actuation elongation over 3 s under 15 kV was measured, with the results presented in the inset of Figure S4b. The samples subjected to 24-hour 5 mbar pressure exhibit almost the same elongation as the control samples (~sea level). The results of elongation and mass change indicate that low pressure has a minor impact on the e-MG.

The temperature of space is another factor that should be considered. The e-MG has three components: silicone rubber, silicone oil, and carbon black, and the tolerable temperature range of them is shown in the Figure S4c. Heat resistance is an exceptional characteristic of silicone rubber, which can withstand over 10,000 hours at 200 °C and short-term use at 300 °C [15]. Silicone rubber is established as a space flight-qualified elastomeric seal material as it works across a wide temperature range and remains flexible at low temperatures [16]. Silicone oil also functions across a broad temperature range, from -55 °C to over 140 °C [17]. In addition, silicone oil exhibits low vapor pressures, low pour points, low compressibility, and low viscosity changes; therefore, it has been used as a space lubricant and in some hydraulic systems in space [18-19]. Carbon black, which consists of nanoparticles, is not considered vulnerable to low temperatures and demonstrates high ignition temperatures exceeding 300 °C [20-21].

The thermal stability of e-MG material was investigated. Differential scanning calorimetry analysis was conducted using the analyser Discovery DSC 25 (TA Instruments, USA) with T-zero aluminium pans. The temperature range for testing spanned from -80 to 300 °C, with a heating rate of 5 °C min^{-1}. The testing results of an e-MG sample (CB-0.50) and a silicone rubber sample (Ecoflex Gel, the matrix component of e-MG) are presented in Figure S4d. The endothermic melting peaks of both e-MG and silicone rubber appear at around -42 °C, which is also observed in other silicone rubber materials [22]. This shows the endothermic peak of e-MG mainly arises from the matrix material, and the e-MG remains at a rubbery phase when the temperature is above -42 °C. The heat flow of e-MG returns to the baseline level, and no major thermal events occur between -38 to 300 °C, revealing the thermal stability in this temperature range. Dynamic mechanical analysis was carried out via a rheometer (Kinexus Pro, NETZSCH, Germany) from 20 to 160 °C with a ramp rate of 3 °C min^{-1}. Oscillatory sweeps were performed at 1 Hz frequency with shear strain of 5 %. As shown in Figure S4e, the storage modulus increases with the increase in temperature, while the loss modulus exhibits a decreasing tendency. This shows the e-MG sample tends to become stiff and the ability to dissipate mechanical energy is weakened in high temperatures [23], which can cause the reduction of actuation performance. Although temperature has an influence on viscoelastic properties, the changes of storage and loss moduli are within 23 and 60 %, respectively, when

the temperature rises from 20 to 160 °C. To verify the ability of e-MG to withstand harsh temperatures, samples were exposed to -20 °C in a freezer, and 60, 100, and 140 °C on a hot plate for 3 hours, while control samples remained at room temperature (20 °C). The actuation elongation in mineral oil at room temperature was then measured using the method described for the insert of Figure S4b. As shown in Figure S4f, the elongation is almost unchanged after exposure to -20 and 60 °C compared to 20 °C, with a slight reduction observed at 100 °C. Although 140 °C causes a significant decline in actuation elongation, a 21 % elongation is still achievable.

Although it is possible to actuate the e-MG in a wide temperature range, the best actuation performance is achieved around room temperature. Traditionally, thermal control techniques have been employed to protect spacecraft [24-26], because some machines and electronics should be operated in a limited temperature range [18, 27], for example, power control unit (-13 to 40 °C), battery (-5 to 25 °C), and solar array drive (-40 to 65 °C). As summarized in Figure S4c, the temperature limit of e-MG components covers the range of common spacecraft electronics. Hence, it is important to reduce the exposure of e-MG robots from extreme temperatures via thermal management during space missions to fully utilize their actuation performance.

Section 3. The contribution of forces in actuation

The electrostatic force (F_E) follows the electric field lines, while the dielectrophoretic force (F_D) aligns with the field gradient direction. In V-shape configured electrodes (Figure 4a), the electric field is oriented horizontally (Figure 4b), and the field gradient is directed vertically (Figure 4c). The actuation behavior of e-MG robots is the result of the interaction of multiple factors. For easy understanding, here, the horizontal displacement of e-MG robot is simplified to the result of F_E, while the vertical displacement is attributed to F_D.

In Figure 4d, the horizontal displacement of the e-MG robot is 1.13 cm (S_1) over 1.2 s (t_1) (from rotation to anchoring) and is attributed to F_E. In Supporting Information Figure S7, the robot is actuated in the same setup, but a 100 Hz alternating voltage is applied, reducing the impact of F_E. Although F_E still exists in this scenario, as indicated by the stretching at the robot's ends, its polarity is oriented in the horizontal direction. The e-MG robot moves 1.58 cm (S_2) vertically in 11 s (t_2) (from rotation to anchoring), which represents the contribution of the weaker force F_D. Assuming the robot's initial velocity is zero and fluidic resistance is neglectable at these velocities, the relationship between displacement (S_1 and S_2), acceleration (a_1 and a_2), time (t_1 and t_2), net forces (F_E and F_D), and mass of robot (m) can be described by the following equations.

$$S_1 = \frac{1}{2} a_1 t_1^2 \tag{S2}$$

$$S_2 = \frac{1}{2} a_2 t_2^2 \tag{S3}$$

$$F_E = m a_1 \tag{S4}$$

$$F_D = m a_2 \tag{S5}$$

By substituting Equation S2 into Equation S4 and Equation S3 into Equation S5, we obtain:

$$F_E = \frac{2 S_1 m}{t_1^2} \tag{S6}$$

$$F_D = \frac{2 S_2 m}{t_2^2} \tag{S7}$$

The ratio of F_E and F_D can be expressed as:

$$\frac{F_E}{F_D} = \frac{S_1 t_2^2}{S_2 t_1^2} = 60.1 \tag{S8}$$

This simple and approximate calculation suggests that the contribution of electrostatic force is around 60 times greater than the dielectrophoretic force when actuating the robots under the V-shape electrodes in Figure 4a.

Section 4. Models of forces and e-MG robot actuation

The electrostatic force is usually used to explain the actuation of charged materials in uniform electric fields. In contrast, the dielectrophoretic force is applied to dielectric materials under non-uniform electric fields. The fundamental physics of both electrostatic and dielectrophoretic forces is Maxwell stress [28-30]; thus, the two forces are interrelated. The e-MG material is composed of conductive networks and a dielectric matrix. The e-MG can be electrically polarized when exposed to an electric field, where free charges accumulate on the surfaces and dipoles rotate to align with the electric field. It results in an electric force acting on the e-MG. From a broad perspective, there is no clear boundary between electrostatic and dielectrophoretic force, but we built a simplified model to provide a clear understanding of the electric force applied on the e-MG. For this analysis, we attribute the contribution of forces based on the uniformity of electric fields.

As shown in Supporting Information Figure S5a, two parallel electrodes are fixed at a distance with dielectric media in between, while a block e-MG is placed in the middle of the two electrodes. The electrodes are applied with opposite voltages of the same magnitude. The structure of this configuration is symmetrical between the left and right sides. We assume the electrode is significantly wider than the e-MG, so the top and bottom electrode regions have minimal impact on the e-MG. Therefore, we focus on the area marked with a dashed box in Figure S5a, and the zoomed-in view is illustrated in Figure S5b. The electric field constructed by parallel plate electrodes is uniform without interruption (Figure S1ai), but the e-MG can distort the surrounding field, resulting in the inhomogeneity (Figure S1aii). The field far away from the e-MG remains uniform, while the field close to the e-MG becomes non-uniform. We assume that the vertical line x_2 is the border between uniform and non-uniform electric fields (Figure S5b).

As the electric field is uniform in region i ($x_1 < x < x_2$), the force in this region (F_i) acting on e-MG towards the left electrode can be expressed as [30]

$$F_i = \frac{1}{2}\varepsilon_i E_i(t)^2 \quad \text{(S9)}$$

where ε_i is the permittivity of dielectric media, and E_i is the electric field in region i. When a voltage is applied, current flows through the system. J_i and J_{ii} are defined as the current densities of region i and ii respectively. E_i is time (t) dependent due to the process of charge migration. According to Gauss's law, the interfacial charge conservation can be expressed as [31]:

$$J_i - J_{ii} = -\frac{\partial}{\partial t}(\varepsilon_i E_i - \varepsilon_{ii} E_{ii}) \quad \text{(S10)}$$

where ε_{ii} is the permittivity of e-MG and E_{ii} is the electric field of region ii. According to Ohm's law, Equation S10 can be expanded as

$$\sigma_i E_i - \sigma_{ii} E_{ii} = -\frac{\partial}{\partial t}(\varepsilon_i E_i - \varepsilon_{ii} E_{ii}) \quad \text{(S11)}$$

where σ_i and σ_{ii} represent the conductivity of the dielectric media and e-MG respectively. When a voltage V_0 is applied to the electrode, the voltages of region i (V_i) and region ii (V_{ii}) can be expressed as

$$V_0 = V_i + V_{ii} = E_i X_i + E_{ii} X_{ii} \quad \text{(S12)}$$

where X_i and X_{ii} is the width of region i and ii respectively, as labeled in Figure S5b. Rearranging Equation S12 results in

$$E_{ii} = \frac{V_0 - E_i X_i}{X_{ii}} \quad \text{(S13)}$$

Substituting Equation S13 to Equation S11 results in

$$E_i(t) = \frac{\sigma_{ii} V_0}{X_{ii}\left(\sigma_i + \frac{\sigma_{ii} X_i}{X_{ii}}\right)}\left(1 - e^{-\frac{t}{\tau}}\right) + \frac{\varepsilon_{ii} V_0}{X_{ii}\left(\varepsilon_i + \frac{\varepsilon_{ii} X_i}{X_{ii}}\right)} e^{-\frac{t}{\tau}} \quad \text{(S14)}$$

$$\tau = \frac{\varepsilon_i + \frac{\varepsilon_{ii} X_i}{X_{ii}}}{\sigma_i + \frac{\sigma_{ii} X_i}{X_{ii}}} \quad \text{(S15)}$$

Substituting Equation S14 to Equation S9 results in

$$F_i = \frac{\varepsilon_i}{2}[\frac{\sigma_{ii} V_0}{X_{ii}\left(\sigma_i + \frac{\sigma_{ii} X_i}{X_{ii}}\right)}\left(1 - e^{-\frac{t}{\tau}}\right) + \frac{\varepsilon_{ii} V_0}{X_{ii}\left(\varepsilon_i + \frac{\varepsilon_{ii} X_i}{X_{ii}}\right)} e^{-\frac{t}{\tau}}]^2 \quad \text{(S16)}$$

Equation S16 demonstrates the contribution of interface charge accumulation, induced by the conductivity of the e-MG, to the resulting electrostatic force on the e-MG.

The electric field in region ii ($x_2 < x < x_3$) is inhomogeneous, and thus the dominant force acted on the e-MG can be approximated as a dielectrophoretic force (F_{ii}) [32-34]:

$$F_{ii} = r\varepsilon_i K \nabla E_{ii}^2 \quad \text{(S17)}$$

$$K = \frac{\varepsilon_{ii} - \varepsilon_i}{\varepsilon_{ii} + 2\varepsilon_i} \quad \text{(S18)}$$

where r is the size factor related to the size of e-MG, K is the Clausius–Mossotti factor, and ∇ is the gradient operator of electric field. A typical voltage distribution in the horizontal direction of the e-MG is shown in Figure S5c. The simulation was conducted via COMSOL Multiphysics based on the model in Figure S5a. The voltage decreases linearly from position x_2 to x_3, indicating the linear non-uniformity of the electric field in region ii. Hence, the electric field is position dependent and can be expressed as:

$$E_{ii} = \frac{dV_{ii}}{dx} \quad \text{(S19)}$$

Substituting Equation S19 to Equation S17 results in

$$F_{ii} = R\varepsilon_i K\nabla\left(\frac{dV_{ii}}{dx}\right)^2 \quad \text{(S20)}$$

After combining the forces in region i and ii (Equations S16 and S20), the electric force ($F_{e\text{-}field}$) acting on e-MG can be expressed as:

$$F_{e-field} = F_i + F_{ii} \quad \text{(S21)}$$

There are two important factors that can influence the intensity of $F_{e\text{-}field}$, i.e., the dielectric constant and conductivity of e-MG. The e-MG samples with high carbon content exhibit large dielectric constant and conductivity; therefore, they experience great electric forces.

To further investigate the morphing behavior of soft material under this force, the charge density throughout the top boundary of e-MG (region ii in Figure S5b) is simulated by COMSOL Multiphysics, as shown in Figure S5d. The highest charge density appears at x_2 and the density reduces dramatically when approaching x_3, indicating that charges tend to accumulate at the sharp edges. According to Coulomb's law, like-charges repel each other. The areas with high charge density experience a large Coulomb force, further leading to the spreading behaviors of the e-MG. This explains why the sections of e-MG with sharp geometry tend to exhibit large morphing. In Supporting Information Figure S6c, we visualized the Maxwell stress tensors on the spreading e-MG profiles based on the demonstration in Figure S6a, b, and Video S4. The Maxwell stress overcomes the internal stress of e-MG material, resulting in stretching towards the counter electrode side (downwards). Meanwhile, the repulsion of like-charges makes Maxwell stress polarize in angled directions, which leads to multidirectional stretching, i.e., spreading. During this process, branches appear on the e-MG due to the non-uniform deformation. Branch structures can not only accumulate more charges but also position themselves closer to the counter

electrodes; therefore, they experience enhanced Maxwell stress. The internal stress of e-MG material—its resistance to deformation—increases with strain. Since both actuation force and internal resistance change dynamically during morphing until equilibrium is reached, the extension curve in Figure S6a shows a non-linear increase over time.

To explain the influence of material elasticity on e-MG actuation, an expanded model of Figure S5a is introduced in Figure S5e, where the e-MG robot is modelled as an elastic conductor, simplified to a spring with zero electrical resistance and conductive plates attached to each end. When paired with the dielectric medium and two outside electrodes, the system can be represented as two capacitors linked by a spring. The outside electrodes are stationary, while the spring between them is free to move. The distances between the plates of Capacitor A and Capacitor B are defined as d_A and d_B, respectively. When the system is powered, the electrical potentials of Capacitor A and Capacitor B are V_A and V_B, respectively. The electric field strength of the two capacitors can be expressed as:

$$E_A = \frac{V_A}{d_A} \tag{S22}$$

$$E_B = \frac{V_B}{d_B} \tag{S23}$$

Due to the series connection, the two capacitors store the same amount of charge, determined as Q. The electrostatic forces exerted on the plate of Capacitor A and Capacitor B are F_A and F_B, respectively (Figure S5e).

$$F_A = \frac{1}{2} Q E_A \tag{S24}$$

$$F_B = \frac{1}{2} Q E_B \tag{S25}$$

Combining Equations S22 and S24 leads to:

$$F_A = \frac{Q V_A}{2 d_A} \tag{S26}$$

Combining Equations S23 and S25 results in:

$$F_B = \frac{Q V_B}{2 d_B} \tag{S27}$$

<u>Translation case:</u>

We assume that $d_A > d_B$, $F_A < F_B$, thus the net force acting on the spring is $F_B - F_A$. If the spring is rigid, it will undergo translation in the direction aligned with the force F_B without

stretching. According to Newton's second law, the instantaneous acceleration can be calculated as:

$$a = \frac{F_B - F_A}{m} \quad \text{(S28)}$$

where *m* is the mass of the robot. The displacement of the spring at time *t* is expressed as:

$$D = \frac{1}{2}at^2 \quad \text{(S29)}$$

During the translation of the spring, the distance d_B decreases from the initial value d_{B0}, while the distance d_A increases from the initial value d_{A0}.

$$d_B = d_{B0} - D \quad \text{(S30)}$$

$$d_A = d_{A0} + D \quad \text{(S31)}$$

Combining Equations S26-31 gives:

$$D = \frac{Q}{4m}\left(\frac{V_B}{d_{B0} - D} - \frac{V_A}{d_{A0} + D}\right)t^2 \quad \text{(S32)}$$

When V_A and V_B are invariable, *D* is a function of *t*. According to Equation S32, the relationship between *D* and *t* is visualized in Figure S5f. The speed of movement increases with *t*. This is because F_B increases and simultaneously F_A reduces as the spring approaches the right electrode, resulting in a stronger net force.

<u>Stretching case:</u>

If the spring is linearly elastic with the spring *k*, it will experience elongation when forces are applied. To minimize complexity, we assume that d_B is 0 and the right end of the spring is adhered to the right electrode. When F_A is exerted at the left side of the spring, the extension can be calculated using Hooke's law.

$$\Delta L = \frac{F_A}{k} \quad \text{(S33)}$$

Substituting Equation S26 into Equation S33 results in:

$$\Delta L = \frac{QV_A}{2kd_A} \quad \text{(S34)}$$

During the stretching process, d_A decreases from the initial value d_{A0}.

$$d_A = d_{A0} - \Delta L \quad \text{(S35)}$$

Substituting Equation S35 into Equation S34 results in:

$$\Delta L = \frac{QV_A}{2k(d_{A0} - \Delta L)} \tag{S36}$$

Equation S36 indicates that equilibrium extension ΔL is a function of k when V_A is constant, as depicted in Figure S5g. The spring with high elastic constant (k) suffers low elongation (ΔL), which reveals the importance of softness in obtaining high actuation extension. As the e-MG exhibits remarkable flexibility, it undergoes both morphing and translation when it is unrestricted.

Section 5. Comparison of actuation in different media

The actuation performance in different media (air and mineral oil) is compared using the method described below. The upper end of the long strip sample was fixed to an insulated electrode, while another insulated electrode was positioned beneath it at a set distance. When exposed to electric fields, the e-MG samples demonstrated stretching actuation, with the maximum elongation over 3 s being recorded. The actuation was conducted in air and mineral oil environments. During actuation, the electric field force deforms the e-MG material by overcoming its elasticity, resulting in a strain (actuation elongation). Based on the measured elongation (strain), stress-strain curves (Supporting Information Figure S2c), and sample size, the stored force can be estimated, which in turn provides insight into the electric field force.

As shown in Supporting Information Figure S11, the mineral oil actuation environment provides higher elongation and stored force compared with air. This is attributed partly to the surface charges dissipating more quickly in air due to leakage currents and humidity. In comparison, dielectric mineral oil can reduce charge leakage, which enhances the electric field distribution and improves the electrostatic force. The higher dielectric constant of mineral oil also results in a stronger dielectrophoretic force, according to Equation 2. In addition, because the maximum applied electric field strength is restricted by the dielectric breakdown strength of the environment, the higher breakdown strength of mineral oil compared to air potentially enables the application of greater voltages to achieve stronger actuation.

Section 6. Comparison with dielectric elastomer actuators

To explore the actuation difference between e-MG and traditional dielectric elastomer actuators (DEAs), a DEA was created by sandwiching a 4 mm thick elastomer (VHB 4910, 3M) between two electrode layers (conductive hydrogel), as shown in Supporting Information Figure S12a. This thickness of the DEA was chosen to approximately match the thickness of the e-MG in Supporting Information Figure S6. Since the e-MG is not pre-stretched before actuation, the elastomer of the DEA remained unstretched in fabrication. The compression in the thickness direction of the DEA during actuation was measured using a laser displacement meter (LK-G152 laser head, Keyence, Japan), and the strain in thickness was calculated. The actuation environment (mineral oil) and applied voltage (20 kV) of DEA and e-MG are identical.

As shown in Figure S12b, the DEA demonstrates 0.033 % strain in thickness (around 1.3 μm displacement), compared with the 286 % strain (about 17800 μm elongation) of e-MG actuation in Figure S6. Although both the DEA and e-MG rely on electrostatic force, their actuation principles are different. The electrostatic force acts on the compliant electrodes of the DEA. The actuation is achieved via the attraction between electrodes, resulting in the compression of elastomer. The electrodes are in direct contact with the dielectric to achieve strong actuation, but this also increases the risk of electrical breakdown. In contrast, the electrostatic force acts on the e-MG material, and the actuation elongation is achieved by the attraction from remote electrodes to e-MG. Electrodes can thus be positioned at a greater distance, minimizing the risk of electrical breakdown while also expanding the actuation space. Their energy loss mechanisms are also different; DEAs typically experience dielectric losses, viscoelastic losses, and leakage currents, whereas e-MG actuation is mainly affected by electric field leakage and charge dissipation.

Supplementary tables and figures

Table S1. Characteristics of e-MG and magnetic robot systems. Bold highlights key characteristics of e-MG robots which are compact, lightweight, suffer low electrical losses and can readily manipulate multiple objects simultaneously.

		e-MG robots	Magnetic field driven robots [35-43]	
Control systems	Field sources	Electrodes [a]	Electromagnets	Permanent magnet
	Mass of field sources	**Low, $O(1\ \text{g})$**	High, $O(10^{4}\ \text{g})$	High, $O(10^{2}\ \text{g})$
	Size of field sources	**Small, $O(1\ \text{mm}^{3})$**	Large, $O(10^{6}\ \text{mm}^{3})$	Large, $O(10^{4}\ \text{mm}^{3})$
	Size ratio of field sources to robots	**Small, $O(10^{-1})$**	Large, $O(10^{6})$	Large, $O(10^{3})$
	Voltage	High, $O(10^{3}\ \text{V})$	**Low, $O(10^{2}\ \text{V})$** [b]	N/A
	Current	**Low, $O(10^{-6}\ \text{A})$**	High, $O(10^{1}\ \text{A})$	N/A
Actuation	Morphing	Stretching; Bending; Twisting; **Spreading**	Stretching; Bending; Curling; Wrapping	
	Locomotion	Jumping; Rolling; Rotating; Climbing; Swimming; Sliding; **Swing**	Jumping; Rolling; Rotating; Climbing; Swimming; Walking; Crawling	
	Simultaneous independent manipulation	Feasible	Unfeasible [c]	
	Operation media	Air; Dielectric liquid	Air; Dielectric liquid; Water	

Note: The symbol *O* represents order of magnitude.
[a] Electrodes can be arbitrary shapes depending on required electric fields. Planar and cylindrical electrodes are employed in this article.
[b] The applied voltage is determined by the required magnetic field and the devices used. The data presented here is sourced from commercial magnetic resonance imaging machines [44-46].
[c] Possible with specially patterned heterogeneous robots [43, 47-49] .

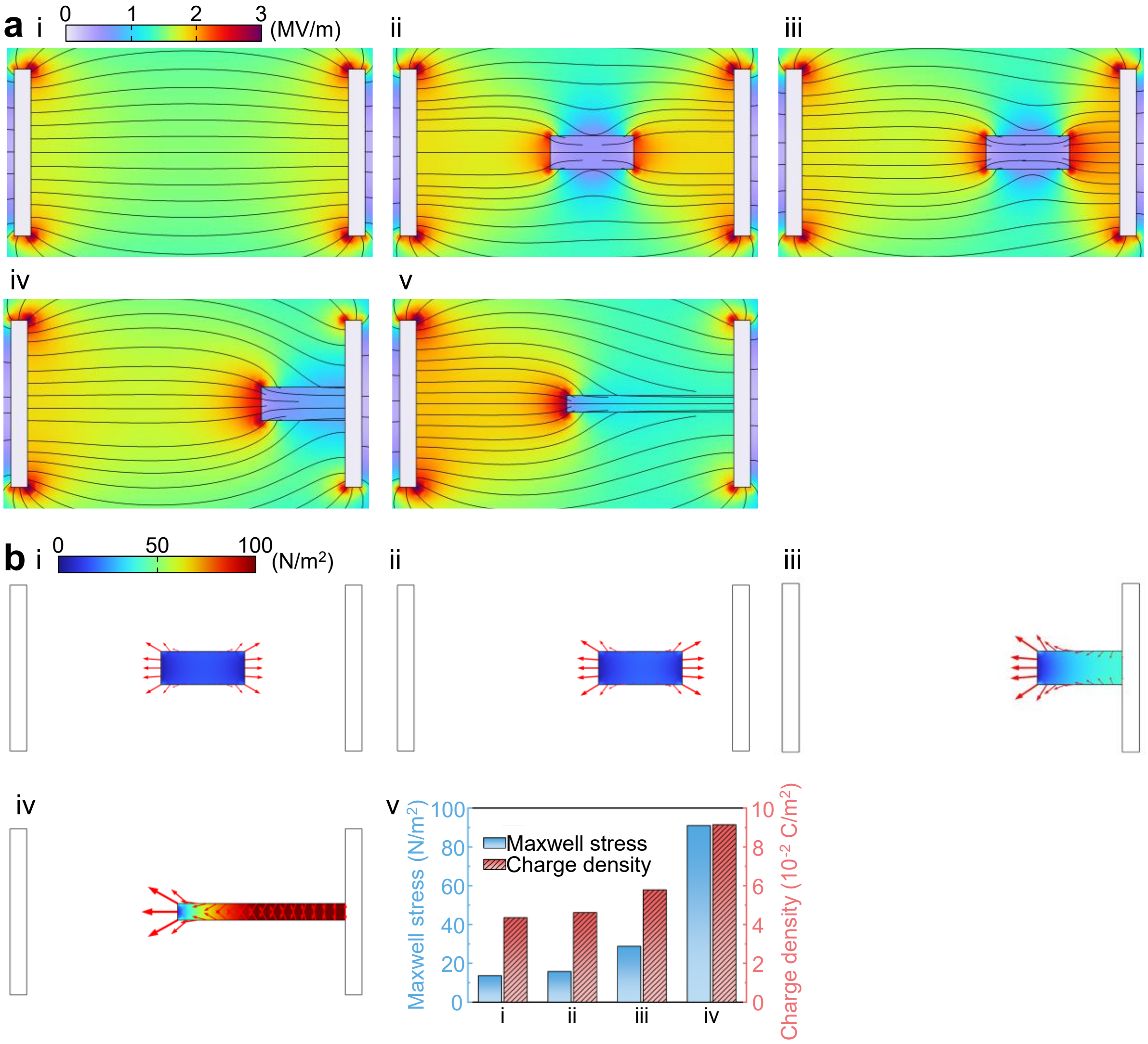

Figure S1. Finite element simulation. a) Simulation of electric field strength distribution with e-MG. Black lines represent electric field lines. (i) The predominantly uniform electric field created by two parallel plate electrodes in a dielectric liquid environment. The electrodes are coated with a dielectric shield layer, with the left and right electrodes subjected to -10 kV and 10 kV, respectively. (ii) A rectangle e-MG robot is placed in the field. (iii) The position of the robot is shifted to the right. (iv) The right side of the robot is in contact with the right electrode. (v) The robot is elongated while the rectangle area is constant. b) Simulation of Maxwell stress magnitude. Red arrows are Maxwell stress tensors on the borderline of the robot. The location and configuration of robot in (i-iv) match (ii-v) in (a). (v) The average values of Maxwell stress and charge density on the perimeter of robot in (i-iv).

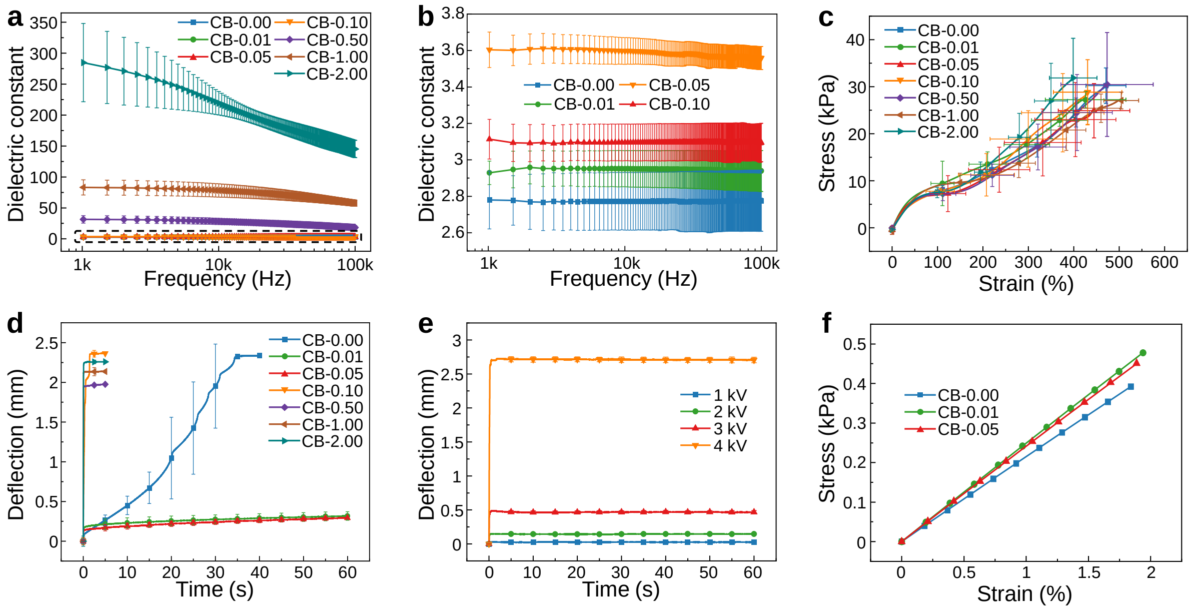

Figure S2. Material and actuation characterization. a) Frequency dependence of dielectric constant. b) Zoomed-in view of dashed box area in (a). c) Stress-strain curves. d) Actuation deflection over time. e) Deflection at different voltages. f) Stress-strain curves at low strain.

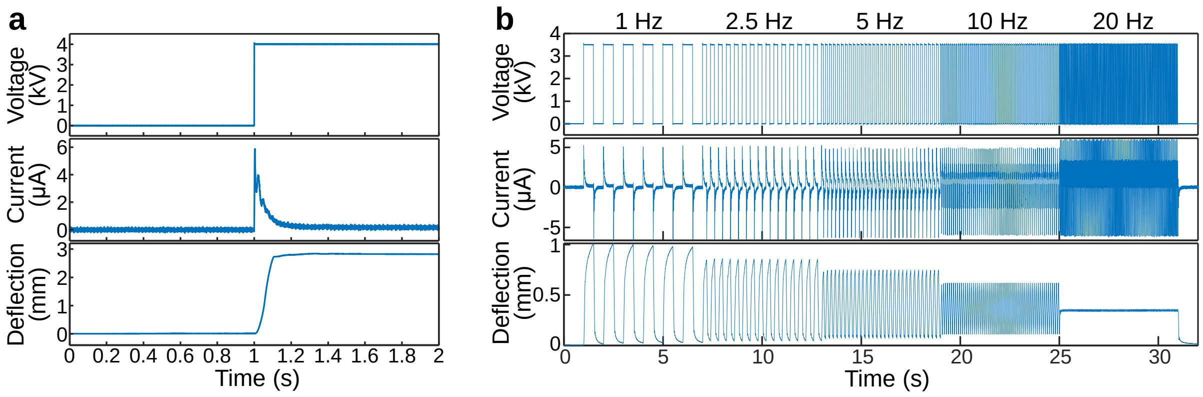

Figure S3. Demonstrations of e-MG actuation in air environment. a) Actuation under a step voltage. b) Actuation under an alternating voltage with changing frequency.

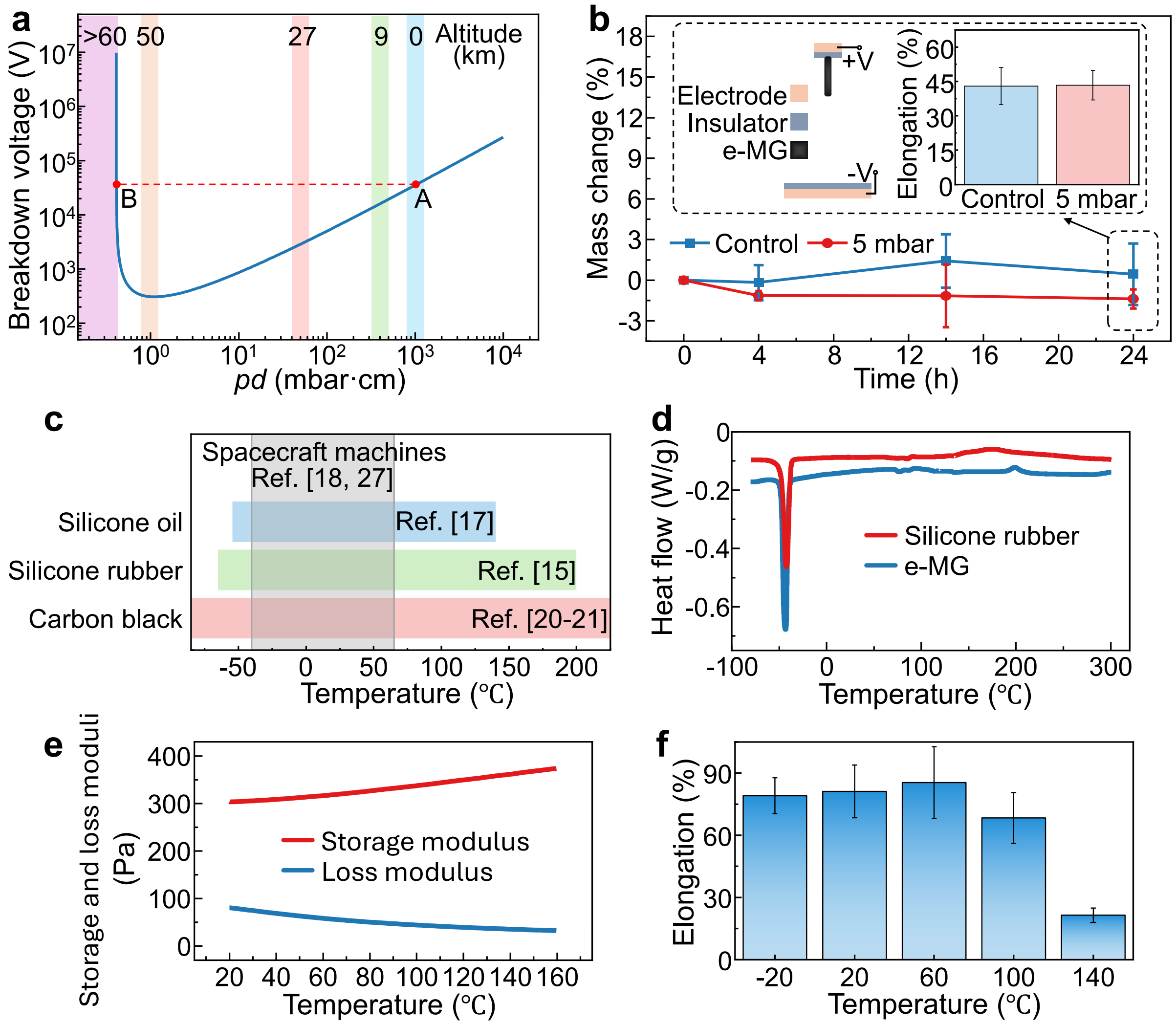

Figure S4. Voltage, pressure, and temperature considerations for space applications. a) Paschen's curve and altitude-related atmospheric pressure. b) Stability of e-MG under low pressure. Insert shows actuation elongation tests in air after vacuuming. c) Operating temperature limits of e-MG components cover those of existing space machines. d) Differential scanning calorimetry analysis of silicone rubber and e-MG. e) Dynamic mechanical analysis of e-MG. f) Actuation elongation in a dielectric liquid after exposure to different temperatures for 3 hours.

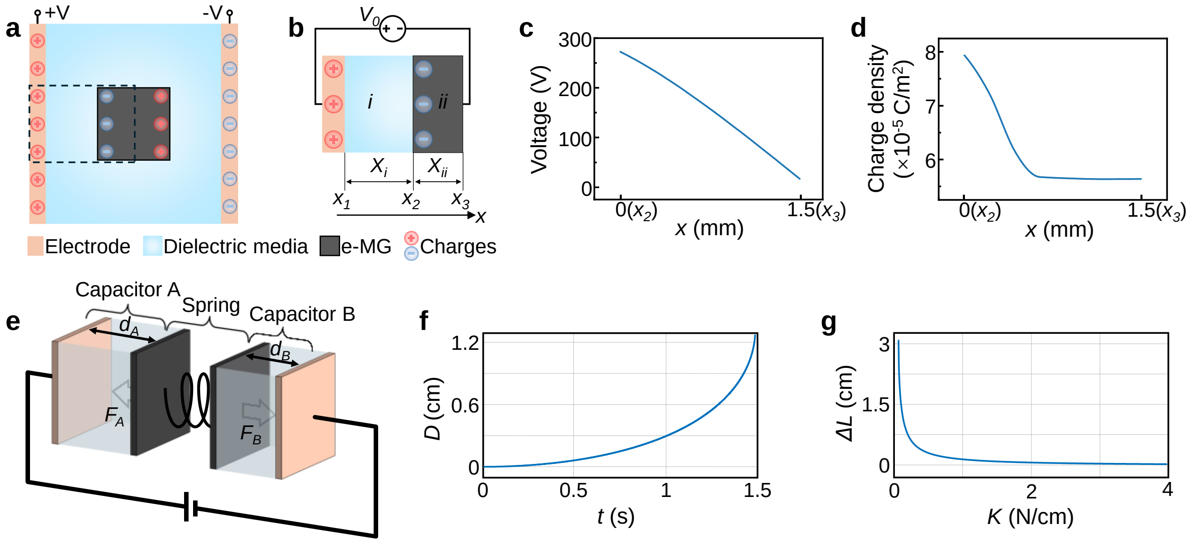

Figure S5. Models illustrating forces and e-MG robot actuation. a) The parallel plate electrode model with e-MG in the middle. b) Zoomed-in illustration of the dashed box area in (a). c) Voltage distribution throughout the e-MG (COMSOL result). d) Charge density distribution throughout the e-MG (COMSOL result). e) Electric circuit model with two capacitors and a spring connecting middle electrodes. f) Translation case; The displacement (*D*) of the rigid spring as a function of time (*t*) (V_A and V_B constant, analytical result). g) Stretching case; Equilibrium elongation of the elastic spring (ΔL) as a function of spring constant (*k*) (d_B = 0 and V_A constant, analytical result).

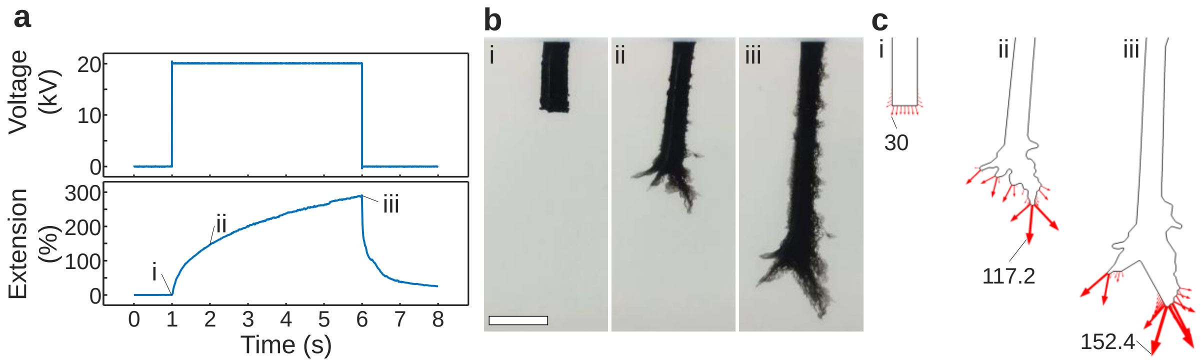

Figure S6. Spreading demonstration of e-MG. a) Applied voltage and measured extension. b) Photographs of the spreading process of e-MG. Scale bar is 5 mm. c) COMSOL simulation of Maxwell stress tensor. The e-MG profiles are depicted according to (b). Red arrows represent the magnitude and polarity of Maxwell stress tensor. The size of arrows is logarithmically related to magnitude. For clarity, the representative values of three arrows are labeled in (c) with the unit of N/m^2. (i-iii) in (a-c) are matched. The actuation was performed in a dielectric liquid environment.

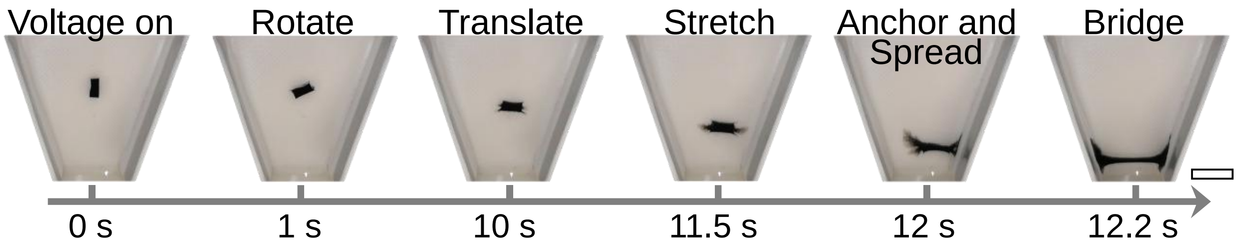

Figure S7. Behaviors of e-MG in V-shape configured electrodes under a 100 Hz alternating voltage. The actuation was performed in a dielectric liquid environment. Scale bar is 1 cm.

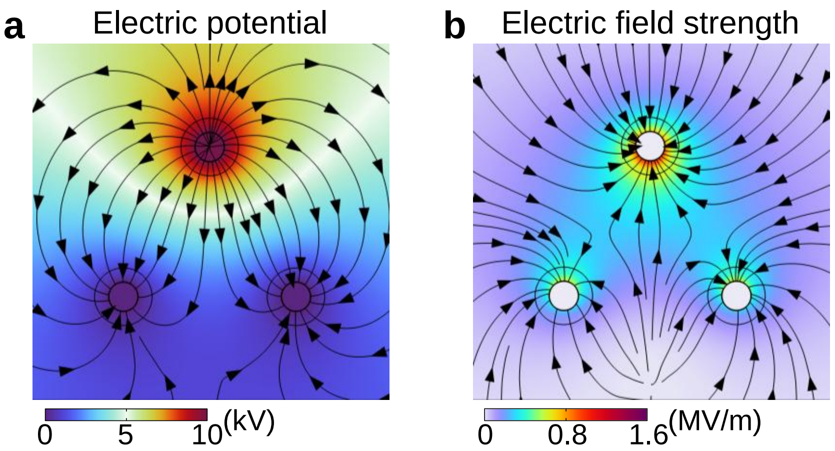

Figure S8. Finite element simulation of electric field when only one cylindrical electrode is energized (lower two electrodes are grounded). a) Electric potential. Black arrows are electric field lines. b) Electric field strength. Black arrows are electric field gradient lines.

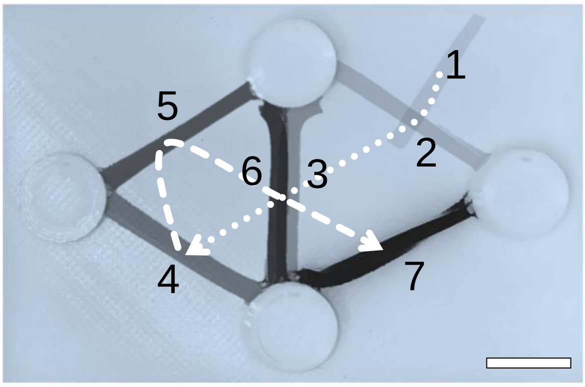

Figure S9. Manipulation of an e-MG robot through a figure of eight path, using four cylindrical electrodes. The actuation environment is a dielectric liquid. Scale bar is 1 cm. Numbers are the locomotion steps of the robot in sequence.

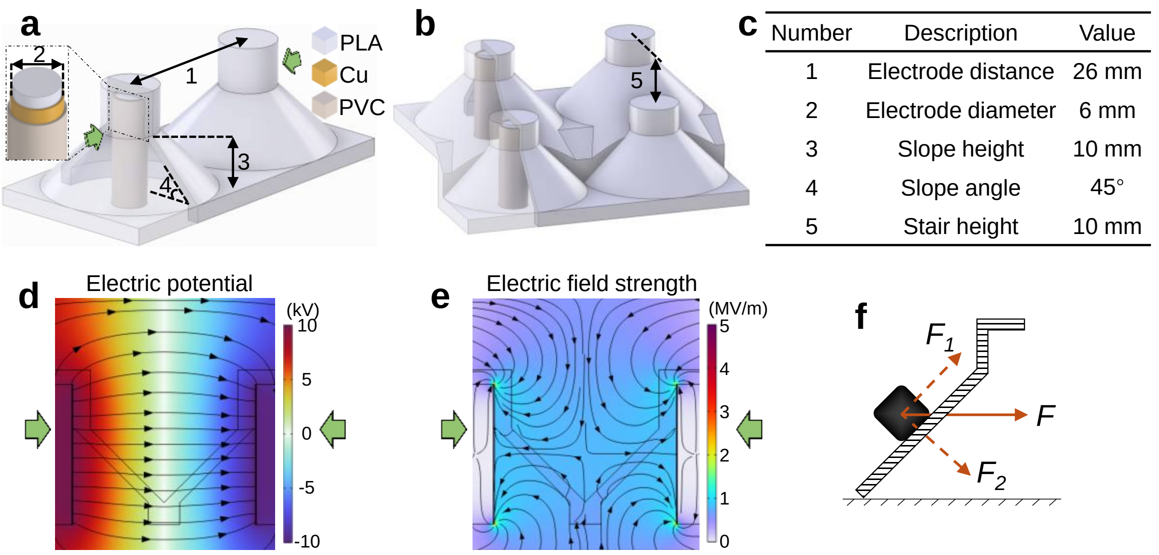

Number	Description	Value
1	Electrode distance	26 mm
2	Electrode diameter	6 mm
3	Slope height	10 mm
4	Slope angle	45°
5	Stair height	10 mm

Figure S10. Conical platforms. a) Diagram of conical platform structure. b) Diagram of two stepped conical platforms. c) Dimensions of the platforms. Numbers (1-5) in (a-c) are matched. d) Electric potential. Black arrows are electric field lines. e) Electric field strength. Black arrows are electric field gradient lines. Green arrows in (d) and (e) align with those in (a). f) Diagram of driving force when an e-MG robot moves on a sloping surface. F is the attraction force from electrode. F_1 and F_2 are component forces of F, which are parallel and perpendicular to the slope respectively.

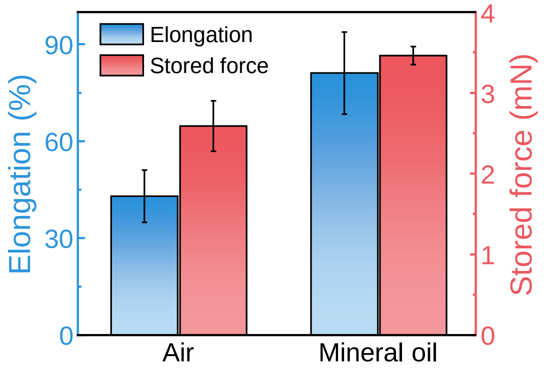

Figure S11. Comparison of actuation elongation and stored force between air and mineral oil actuation environments.

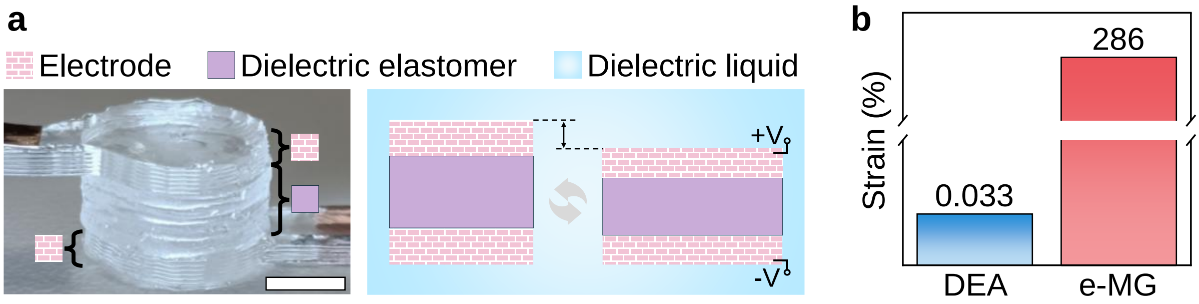

Figure S12. Comparison of e-MG with a dielectric elastomer actuator. a) Photograph and diagram of the DEA. Electrode is conductive hydrogel. Scale bar is 5 mm. b) Comparison of actuation strain between DEA and e-MG.

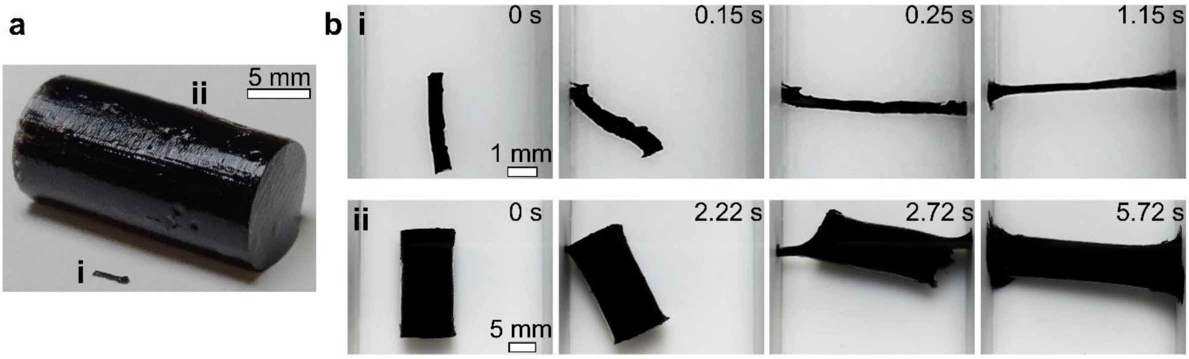

Figure S13. Demonstration of scalability. a) Relative sizes of the two robots. (i) 0.25 × 0.5 × 3 mm, and (ii) 10 (diameter) × 20 (length) mm, show 4189 times volume (and mass) difference. b) Actuation of the two robots between insulated parallel plate electrodes in a dielectric liquid environment. Robots (i) and (ii) match in (a) and (b).

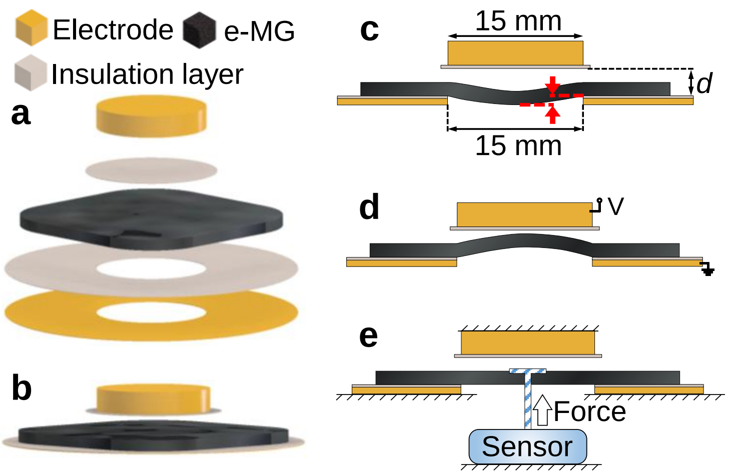

Figure S14. Diagram of actuation deflection and force measurement setup. a) Exploded view. b) Front view. c) Half section view in unactuated state. Red dash lines and arrows indicate the sagging of e-MG membrane. *d* represents the distance between two electrodes. d) Half section view in actuated state. e) Actuation force measurement method.

Diagram PLA Cu PVC	Number	Description	Value
**a** 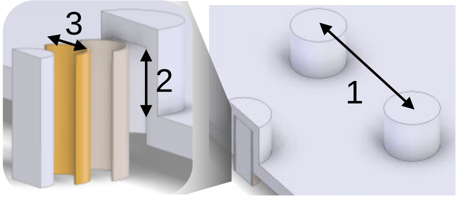	1	Electrode distance	30 mm
	2	Electrode height	6 mm
	3	Electrode diameter	6 mm
**b** 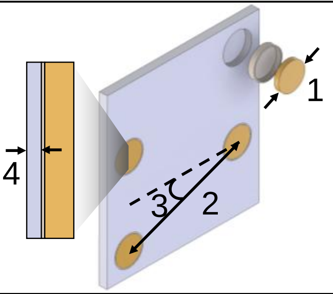	1	Electrode diameter	6 mm
	2	Electrode distance	26 mm
	3	Angle between electrodes	20°
	4	Platform thickness	0.5 mm
**c** 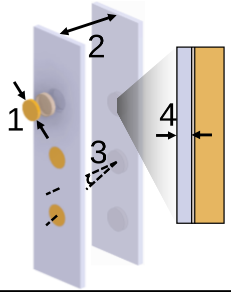	1	Electrode diameter	6 mm
	2	Channel width	20 mm
	3	Angle between electrodes	20°
	4	Platform thickness	0.5 mm

Figure S15. Diagram and size of the electrode configurations used. a) Cylindrical electrode configuration. b) Vertical wall with flat surface and planar electrodes. c) Channel configuration with embedded planar electrodes.

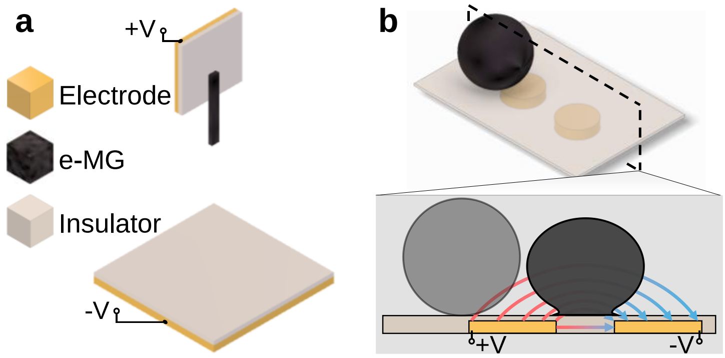

Figure S16. Setup diagrams of demonstrations in air. a) Setup for morphing and gripping demonstration. b) Setup for manipulation demonstration. Driven by the electric field generated between the two energized electrodes, the e-MG spherical robot rolls forward while undergoing deformation.

List of Supplemental Videos

Video S1: Demonstration of the deformability of e-MG robots
a) An e-MG gymnast swinging along a ceiling
b) An e-MG sail jumping over a gap
c) An e-MG robot delivering cargo through a channel

Video S2: Demonstration of e-MG actuation
a) Under a step voltage
b) Under an alternating voltage

Video S3: Response of e-MG under a V-shape electrode configuration
a) Under direct voltage
b) Under an alternating voltage

Video S4: Spreading morphing of e-MG

Video S5: Manipulation of e-MG robots with cylindrical electrodes
a) Demonstration on a three-electrode platform
b) Figure of eight path
c) Straight route
d) Curved route
e) Simultaneous and independent manipulation of two e-MG robots

Video S6: Locomotion on a conical surface
a) One pair of conical surfaces
b) Two pairs of stepped conical surfaces

Video S7: An e-MG robot moving up a vertical flat surface

Video S8: An e-MG gymnast moving through a vertical channel

Video S9: Demonstrations of morphing, gripping, and manipulation in air
a) Morphing of an e-MG strip with branches
b) Morphing of a kirigami e-MG
c) A frog tongue inspired e-MG gripper
d) Manipulation of e-MG spherical robots

Video S10: Demonstration of scalability

References

[1] B. P. Sahoo, K. Naskar, R. N. P. Choudhary, S. Sabharwal, D. K. Tripathy, *Journal of Applied Polymer Science* **2012**, 124, 678.
[2] B.-J. Lee, H.-M. Yoo, *Polymers* **2024**, 16, 933.
[3] M. Panahi-Sarmad, B. Zahiri, M. Noroozi, *Sensors and Actuators A: Physical* **2019**, 293, 222.
[4] Y. Dou, S. Sun, S. Lu, W. Yao, D. Guan, *RSC Advances* **2022**, 12, 32448.
[5] A. A. Martins, M. J. Pinheiro, *Physics Procedia* **2011**, 20, 112.
[6] E. Sili, F. Koliatene, J. P. Cambronne, presented at *2011 Annual Report Conference on Electrical Insulation and Dielectric Phenomena*, 16-19 Oct, **2011**. DOI: 10.1109/CEIDP.2011.6232695.
[7] P. Mathew, J. George, S. Mathews T, P. J. Kurian, *AIP Advances* **2019**, 9, 025215.
[8] C. R. Rao, G. R. G. Raju, *Journal of Physics D: Applied Physics* **1971**, 4, 494.
[9] E. Husain, R. S. Nema, *IEEE Transactions on Electrical Insulation* **1982**, EI-17, 350.
[10] A.-L. Paul, R. Ferl, *Gravitational and Space Biology* **2006**, 19, 3.
[11] E. Yiğit, R. R. Garcia, in *Reference Module in Earth Systems and Environmental Sciences*, Elsevier, **2018**. https://www.sciencedirect.com/science/article/pii/B9780124095489116112.
[12] D. S. A. Hewaralalage, I. Sherrington, N. Renevier, M. Bernabei, A. Ghanbari, *arXiv preprint arXiv:2308.09509* **2023**.
[13] E. V. Zaretsky, *Tribology International* **1990**, 23, 75.
[14] M. Lizcano, T. S. Williams, E.-S. E. Shin, D. Santiago, B. Nguyen, *Materials* **2022**, 15, 8121.
[15] S. C. Shit, P. Shah, *National academy science letters* **2013**, 36, 355. https://doi.org/10.1007/s40009-013-0150-2.
[16] H. C. I. deGroh, C. C. Daniels, J. A. Dever, S. K. Miller, D. L. Waters, J. R. Finkbeiner, P. H. Dunlap, B. M. Steinetz, *NASA Technical Memorandum* **2010**, 20100029591. https://ntrs.nasa.gov/citations/20100029591.
[17] Safety Data Sheet: Silicone oil, Sigma-Aldrich. https://www.sigmaaldrich.com/GB/en/sds/aldrich/378356?userType=anonymous.
[18] R. L. Fusaro, M. M. Khonsari, *NASA Technical Memorandum* **1992**, 105198. https://ntrs.nasa.gov/api/citations/19920022596/downloads/19920022596.pdf.
[19] T. Kałdoński, P. P. Wojdyna, *Journal of KONES Powertrain and Transport* **2011**, 18, 163.
[20] N. Cornea, *Revist De Chim* **2011**, 62, 923.
[21] S. Nakayama, *Scientific Reports* **2021**, 11, 9574.
[22] Application brief, Hitachi High-Tech Science Corporation. https://www.hitachi-hightech.com/file/global/pdf/products/science/appli/ana/thermal/application_TA_018e.pdf.
[23] E. Porte, S. Eristoff, A. Agrawala, R. Kramer-Bottiglio, *Soft Robotics* **2023**, 11, 118.
[24] D. W. Hengeveld, M. M. Mathison, J. E. Braun, E. A. Groll, A. D. Williams, *HVAC&R Research* **2010**, 16, 189.
[25] J. Miao, Q. Zhong, Q. Zhao, X. Zhao, *Spacecraft thermal control technologies*, Springer, **2021**.
[26] T. D. Swanson, G. C. Birur, *Applied Thermal Engineering* **2003**, 23, 1055.
[27] J. Plante, B. Lee, *NASA document* **2005**. https://ntrs.nasa.gov/citations/20060013394.
[28] T. M. Minteer, *IEEE Transactions on Dielectrics and Electrical Insulation* **2014**, 21, 64.
[29] X. Wang, X.-B. Wang, P. R. C. Gascoyne, *Journal of Electrostatics* **1997**, 39, 277.
[30] Z. Suo, *Acta Mechanica Solida Sinica* **2010**, 23, 549.

[31] Y. Xu, J. Wen, E. Burdet, M. Taghavi, *Nature Communications* **2025**, 16, 1174.
[32] B. Çetin, D. Li, *Electrophoresis* **2011**, 32, 2410.
[33] T. B. Jones, *Electromechanics of Particles*, Cambridge University Press, Cambridge **1995**.
[34] R. Pethig, *Journal of The Electrochemical Society* **2017**, 164, B3049.
[35] Y. Cheng, K. H. Chan, X.-Q. Wang, T. Ding, T. Li, C. Zhang, W. Lu, Y. Zhou, G. W. Ho, *Advanced Functional Materials* **2021**, 31, 2101825.
[36] Y. Yang, S. Yuan, H. Ren, *Advanced Functional Materials* **2024**, 34, 2311981.
[37] W. Hu, G. Z. Lum, M. Mastrangeli, M. Sitti, *Nature* **2018**, 554, 81.
[38] Y. Kim, G. A. Parada, S. Liu, X. Zhao, *Science Robotics* **2019**, 4, eaax7329.
[39] Y. Ko, S. Na, Y. Lee, K. Cha, S. Y. Ko, J. Park, S. Park, *Smart Materials and Structures* **2012**, 21, 057001.
[40] M. Sun, C. Tian, L. Mao, X. Meng, X. Shen, B. Hao, X. Wang, H. Xie, L. Zhang, *Advanced Functional Materials* **2022**, 32, 2112508.
[41] D. Li, F. Niu, J. Li, X. Li, D. Sun, *IEEE Transactions on Industrial Electronics* **2020**, 67, 4700.
[42] J. Zhao, C. Xin, J. Zhu, N. Xia, B. Hao, X. Liu, Y. Tan, S. Yang, X. Wang, J. Xue, Q. Wang, H. Lu, L. Zhang, *Advanced Materials* **2024**, 36, 2312655.
[43] E. Diller, J. Giltinan, M. Sitti, *The International Journal of Robotics Research* **2013**, 32, 614.
[44] Product data: Magnetom Altea 1.5 Tesla, Healthineers. https://doclib.siemens-healthineers.com/rest/v1/view?document-id=600566.
[45] Product data: Ingenia Ambition 1.5T, Philips. https://www.philips.co.uk/c-dam/b2bhc/gb/resource-catalog/landing/brightontender/mr-ingenia-ambition-1-5-t-product-specifications.pdf.
[46] Product data: Hitachi Echelon 1.5T, Clinical Imaging Systems. https://www.scribd.com/document/719601562/Hitachi-Echelon-1-5T-product-data.
[47] S. Floyd, E. Diller, C. Pawashe, M. Sitti, *The International Journal of Robotics Research* **2011**, 30, 1553.
[48] Y. Lee, F. Koehler, T. Dillon, G. Loke, Y. Kim, J. Marion, M.-J. Antonini, I. C. Garwood, A. Sahasrabudhe, K. Nagao, X. Zhao, Y. Fink, E. T. Roche, P. Anikeeva, *Advanced Materials* **2023**, 35, 2301916.
[49] C. Wang, T. Wang, M. Li, R. Zhang, H. Ugurlu, M. Sitti, *Science Advances* **2024**, 10, eadq1951.
